# System inflammation response index: a novel inflammatory indicator to predict all-cause and cardiovascular disease mortality in the obese population

**DOI:** 10.1186/s13098-023-01178-8

**Published:** 2023-10-11

**Authors:** Fanliang Kong, Junhao Huang, Chunhua Xu, Tingyuan Huang, Grace Wen, Wenke Cheng

**Affiliations:** 1 University Medical Center of Göttingen, Georg-August University, Göttingen, Germany; 2 Department of Metabolic and Bariatric Surgery, The First Affiliated Hospital of Jinan University, Guangzhou, Guangdong Province China; 3 Department of Plastic Surgery, The First Affiliated Hospital of Jinan University, Guangzhou, Guangdong Province China; 4 Department of Gastrointestinal Surgery, The First Affiliated Hospital of Jinan University, Guangzhou, Guangdong Province China; 5 Department of Recuperation, Lintong Rehabilitation, and Recuperation Center, Xian, Shaanxi China; 6 Department of Oncology, Affiliated Cancer Hospital & Institute of Guangzhou Medical University, Guangzhou, China; 7 Medical Faculty, University of Leipzig, Leipzig, Germany

**Keywords:** Inflammation, System inflammation response index, Systemic immune inflammatory index, Cardiovascular disease, All-cause mortality, Mortality

## Abstract

**Aim:**

This study aims to investigate the relationship between two novel inflammatory markers, namely, the Systemic Inflammatory Response Index (SIRI) and the Systemic Immune Inflammatory Index (SII), as well as the all-cause and cardiovascular disease (CVD) mortality in the obese population.

**Materials and methods:**

We conducted a prospective cohort study based on the data of 13,026 obese adults (age ≥ 18 years) from the National Health and Nutrition Examination Survey (NHANES) from 1999 to 2014 and followed until December 2019. SIRI was calculated by the formula: (neutrophil count × monocyte count) / lymphocyte count, while that of SII was: (platelet count × neutrophil count)/lymphocyte count. The association of SIRI and SII with all-cause and CVD mortality was evaluated using Cox regression. In addition, the nomogram was performed to predict 10-year survival probability.

**Results:**

During a median follow-up of 137 months, 1959 and 553 all-cause and CVD deaths were recorded, respectively. Spearman correlation analysis indicated that SIRI and SII were unrelated to almost all baseline characteristics (r < 0.15). Multivariate Cox regression models displayed that each standard deviation (SD) increase in SIRI was associated with a 16% (HR 1.16; 95% CI 1.09–1.24) and 22% (HR 1.22; 95% CI 1.10–1.36) increase in the risk of all-cause and CVD mortality, respectively. Likewise, every SD increase in SII was correlated with a 9% (HR 1.09; 95% CI 1.02–1.16) and 14% (HR 1.14; 95% CI 1.04–1.26) increase in the risk of all-cause and CVD mortality, respectively. The predictive value of SIRI for all-cause and CVD mortality (AUC = 0.601 and 0.624) exceeded that of SII (AUC = 0.528 and 0.539). Moreover, the nomogram displayed a substantial predictive value for 10-year survival (AUC = 0.847) with sensitivity and specificity exceeding 75%.

**Conclusions:**

In the obese population, SIRI and SII are independent risk factors for all-cause and CVD mortality. Notably, the predictive ability of SIRI for both all-cause and CVD mortality significantly outperforms that of SII, suggesting that SIRI is a more valuable marker of inflammation.

**Supplementary Information:**

The online version contains supplementary material available at 10.1186/s13098-023-01178-8.

## Introduction

The World Health Organization (WHO) defines obesity as “abnormal or excessive fat accumulation that presents a risk to health” [1]. Body mass index (BMI) is a widely used and universally accepted anthropometric method to classify overweight and obesity, which has been used by WHO, the Centers for Disease Control and Prevention (CDC) and the National Institutes of Health (NIH) as a standard to statistically record obesity [2]. Globally, the prevalence of obesity, which is considered as a chronic disease, is on the rise [3, 4]. According to a report from WHO, approximately 60% of adults and almost one in three children in Europe, including 29% of boys and 27% of girls, are affected by overweight and obesity [5]. Simultaneously, several epidemiological studies confirmed a link between obesity and a broad range of chronic illnesses, including Non-Alcoholic Fatty Liver (NAFL), cardiovascular disease (CVD), diabetes mellitus, certain cancers, musculoskeletal disorders, chronic kidney disease, and mental health conditions [6–12]. It has been suggested that the presence of chronic systemic inflammation is caused by a possible correlation between obesity and these health risks [13, 14]. Accumulated fat cells can overproduce adipokines, which release pro-inflammatory cytokines that trigger an inflammatory response [15, 16]. As a result, obese individuals tend to persist in a state of chronic low-grade inflammation, eventually contributing to the development of various chronic diseases that can potentially lead to death.

CVD is regarded as the leading cause of death worldwide, taking the lives of about 17.9 million people each year [17]. It can be triggered by several risk factors, including dyslipidemia, hypertension, insulin resistance, hypercoagulability, and inflammatory responses [18]. It is widely acknowledged that individuals with chronic inflammation or obesity show a substantially higher susceptibility to cardiovascular disease compared to the general population, which emphasizes the necessity for developing innovative markers capable of detecting chronic inflammatory status to aid in the prediction of cardiovascular disease risk among the obese population [19, 20]. The systemic inflammation response index (SIRI) and systemic immune inflammation index (SII), which are composite indices incorporating three distinct subsets of white blood cells and platelets, provide novel insights into the interplay of thrombocytosis, inflammation, and immunity [21–24]. Recently, the associations of increased SIRI and SII levels with the increased risks of all-cause and CVD mortality has been found in the general population and individuals with hypertension [21, 25–27]. However, the predictive value of SIRI and SII for all-cause and cardiovascular disease mortality in the obese population remains unknown. Therefore, this study examines the association between SIRI and SII levels and the risks of all-cause and CVD mortality in the obese population.

## Materials and methods

### Study design and population

This study applied data from the National Health and Nutrition Examination Survey (NHANES) conducted between 1999 and 2014. Using a complex stratified multistage probability design, NHANES is a survey of the civilian non-institutionalized population in the United States [28]. The survey consists of interviews, physical examinations in the home or at the mobile examination centers (MECs), and laboratory tests and is conducted every 2 years. NHANES was carried out by the National Center for Health Statistics of the Centers for Disease Control and Prevention (CDC) and approved by the NHANES Institutional Review Board, with written informed consent obtained from all the participants.

A total of 18,246 obese adults (age ≥ 18 years) were enrolled in the NHANES survey between 1999 and 2014, among whom 3197 participants were excluded due to the inability to calculate SIRI or SII data. 13,026 participants were included after excluding certain groups. Specifically, 492 pregnant women, 1260 individuals with cancer, and 11 individuals with no follow-up data were excluded. 260 individuals who died within two years were also excluded to minimize the risk of reverse causation bias. The flow chart of the study is presented in Additional file 1: Figure S1.

### Definition of SIRI

The Beckman Coulter MAXM instrument at the MECs produced complete blood cell counts on blood specimens. Detailed specimen collection and processing instructions are well described in the *Laboratory/Medical Technologist Procedure Manual* [29]. The following formula was adopted for calculating SIRI and SII [24, 30]:

$${\text{SIRI}}\, = \,\left( {{\text{neutrophil count}}\, \times \,{\text{monocyte count}}} \right)/{\text{lymphocyte count}}$$

$${\text{SII}}\, = \,\left( {{\text{platelet count}}\, \times \,{\text{neutrophil count}}} \right)/{\text{lymphocyte count}}$$

### Identification of obesity

Body mass index is the ratio of body weight (kg) to height (m) squared, with the result being rounded to two decimal places. In line with the WHO guidelines for Western populations, a BMI ≥ 30 kg/m^2^ is considered obesity [1, 3].

### Definition of outcomes

The outcomes of this study were all-cause and CVD mortality. Mortality data was determined by linking to the National Mortality Index until December 2019. All-cause mortality refers to deaths from all causes. CVD mortality is a general term for deaths from various heart diseases. CVD mortality is coded as I00–I09, I11, I13, and I20–I51 by the International Classification of Diseases, 10th Revision (ICD-10).

### Other variables of interest

Baseline characteristics of participants, including age, sex, race, education levels, poverty-income ratio, smoking and drinking habits, medical history, and medication use, were collected through standardized questionnaires. The participant’s medical conditions were self-reported and verified through medical records provided by a healthcare professional or physician. Biochemical parameters were measured by following a rigorous procedure, and the details are provided in the *NHANES Procedures Manual for Laboratory/Medical Technologists* [29]. In order to facilitate data integration, the following variables were further classified:

(i). Race: non-Hispanic white people, non-Hispanic black people, Mexican Americans, or other races.
(ii). Educational levels: Less than 9th grade, 9th–11th grade/high school or equivalent, college graduate or above.
(iii). Smoking status: Never (< 100 cigarettes/lifetime), former smoker (≥ 100 cigarettes/lifetime and currently not smoking), or current smoker (> 100 cigarettes/lifetime and currently smoking some days or every day) [31].
(iv). Drinking status: Never (< 12 drinks/lifetime), former drinker (≥ 12 drinks/lifetime but not in the past year), current light/moderate drinker (≤ 1 drink/day for women and ≤ 2 drinks/day for men in the past year), or current heavy drinker (> 1 drink/day for women and > 2 drinks/day for men in the past year) [32].

### Statistical analysis

To provide nationally representative estimates, we selected appropriate weights (MEC weights) for data analysis to account for oversampling, non-response, and non-coverage. A detailed description of the weighting methods is presented on the NHANES website (https://wwwn.cdc.gov/nchs/nhanes/tutorials/Module3.aspx). The baseline demographic characteristics were presented as weighted means (95% confidence interval; CI) for continuous variables and weighted percentage (95% CI) for categorical variables.

The Chi-square test was conducted to explore differences between categorical variables. In the case of non-normal distributions, the Mann–Whitney U and Kruskal–Wallis tests were adopted for comparisons of two groups and multiple groups, respectively. Spearman's rank correlation coefficient was employed to determine any association between SIRI and baseline variables, including age, sex, race, poverty income ratio (PIR), BMI, alanine aminotransferase (ALT), aspartate aminotransferase (AST), total cholesterol (TC), high-density lipoprotein cholesterol (HDL-C), estimated glomerular filtration rate (eGFR), and medical history. A correlation coefficient (r) of less than 0.1, 0.1–0.2, 0.2–0.3, 0.3–0.6, and 0.6–1.0 is considered none/negligible correlation, weak correlation, moderate correlation, strong correlation, and very strong correlation, respectively [33].

In addition, quartiles were utilized to categorize the levels of SIRI and SII. A standard normal distribution of SIRI and SII (mean = 0, standard deviation (SD) = 1) was created by standardizing the Z-scores. Using Kaplan–Meier analysis and log-rank tests, cumulative hazard risks at the quartile level of SIRI during the observation period were analyzed. Hazard ratios (HRs) and 95% confidence intervals (CIs) between SIRI and SII and all-cause and CVD mortality were calculated using Cox proportional hazards models. P values for the trend were calculated. Furthermore, baseline variables with a P-value of ≤ 0.1 were considered candidate predictors for the multivariate regression model. In addition, confounding covariates were categorized and progressively added to the different models.

Subgroup analyses were performed, with data stratified by clinical characteristics, including sex (male, female), age (< 60, ≥ 60 years), body mass index (30–34.9, 35–39.9, ≥ 40 kg/m^2^), race (non-Hispanic white people, non-Hispanic black people, Mexican Americans, and other), cardiovascular diseases (no/yes), diabetes mellitus (no/yes), hyperlipidemia (no/yes), hypertension (no/yes), smoking (never, former, current), and medication use (no/yes). Then, p-values were obtained for the interactions.

A generalized additive model (GAM) with a spline smoothing function was adopted for assessing the dose–response relationship between SIRI and SII levels and the risk of all-cause and CVD mortality. GAM was performed using both log-transformed and non-transformed methods. The logarithm of hazard ratio (log HR) was converted to an HR using an anti-logarithm. Under these conditions, a log (HR) of 0 and 1 implies a nonsignificant HR of 1 and 2.71-fold HR, respectively [34]. Nonlinear P-values were obtained according to the log-likelihood ratio test [35]. If a nonlinear association was observed, threshold effect analysis was based on a two-piecewise linear regression model. This facilitated the calculation of the inflection point on the smoothing curve, indicative of a significant alteration in the relationship between SIRI, SII levels, and the risk of all-cause and CVD mortality [36].

We plotted the receiver operating characteristic (ROC) curves to determine the predictive value of SII and SIRI for all-cause and CVD mortality. The area under the ROC curve (AUC) was calculated to assess the model's predictive power. The models with better predictive power were screened out by comparing their predictive values. Then, the baseline variables were tested by univariate analysis and multivariate Cox regression models. Meanwhile, nomograms were constructed to predict the risk of mortality. In line with the regression coefficients, the value of each included variable is consistent with a point on the top line of the total score. The total score is equivalent to the sum of the scores of all the variables for each patient. The total score and prognosis relationship are shown at the bottom of the nomogram. Subsequently, a calibration curve was plotted to evaluate the agreement between the predicted and actual probabilities of the nomogram. The diagonal line represents the best prediction as the reference line. Besides the calibration curve, we also used the AUC and Nagelkerke’s R^2^ to assess the model's calibration accuracy [37]. Moreover, the decision curve analysis was performed by estimating the net benefit at different threshold probabilities, aiming to determine the suitability of the established nomograms for clinical application.

All the analyses were performed with the statistical package R (http://www.R-project.org, R Foundation) and EmpowerStats (version 4.2.0, www.R-project.org, X&Y Solutions, Inc., Boston, MA). Bilateral P values less than 0.05 were of statistical significance.

## Results

From 1999 to 2014, 13,026 obese adults were enrolled in NHANES. Their weighted mean age was 46.2 (standard deviation: 17.3) years, with 47% being male. There were no statistically significant differences in aspartate aminotransferase, prevalence of hyperlipidemia, or history of alcohol consumption among the SIRI quartiles. Similarly, no significant differences were observed in the associations of eGFR, TC, diabetes, cardiovascular disease, hypertension, smoking, alcohol consumption, and drug use among the SII quartiles. Detailed weighted overall demographic characteristics are displayed in Table 1 and Additional file 1: Table S1.

**Table 1:** Survey-weighted baseline characteristics of the obese population in NHANES from 1999 to 2014 according to SIRI quartiles (N = 13,026, representing 63,479,085 individuals with obesity)

	Q1 (< 0.71)	Q2 (0.71–1.05)	Q3 (1.05–1.50)	Q4 (> 1.50)	P-value
Participants, n	3255	3254	3252	3265	
Monocytes (× 10^3^ cells/ml)	0.43 (0.41, 0.44)	0.51 (0.51, 0.52)	0.59 (0.58, 0.60)	0.72 (0.71, 0.73)	< 0.001
Lymphocytes (× 10^3^ cells/ml)	2.45 (2.38, 2.53)	2.30 (2.27, 2.33)	2.25 (2.21, 2.28)	2.08 (2.05, 2.11)	< 0.001
Neutrophils (× 10^3^ cells/ml)	3.11 (3.07, 3.16)	4.01 (3.96, 4.05)	4.76 (4.71, 4.81)	6.14 (6.07, 6.22)	< 0.001
Age (years)	44.9 (44.3, 45.6)	45.3 (44.7, 45.9)	46.6 (45.9, 47.3)	47.7 (47.0, 48.5)	< 0.001
Poverty income ratio	2.7 (2.6, 2.8)	2.9 (2.8, 3.0)	2.9 (2.8, 3.0)	2.8 (2.7, 2.9)	< 0.001
Body mass index (Kg/m^2^)	35.3 (35.0, 35.6)	35.4 (35.1, 35.6)	35.8 (35.5, 36.0)	36.4 (36.1, 36.8)	< 0.001
HDL (mmol/L)	1.3 (1.2, 1.3)	1.2 (1.2, 1.2)	1.2 (1.2, 1.2)	1.2 (1.2,1.2)	< 0.001
TC (mmol/L)	5.2 (5.2, 5.3)	5.2 (5.1, 5.2)	5.1 (5.1, 5.2)	5.1 (5.0, 5.1)	< 0.001
eGFR	98.7 (97.6,99.7)	96.3 (95.3,97.2)	94.2 (93.2,95.3)	92.3 (91.2, 93.4)	< 0.001
ALT (U/L)	27.7 (26.9, 28.5)	29.4 (28.6, 30.3)	29.3 (28.4, 30.3)	29.2 (28.4, 30.0)	0.023
AST (U/L)	25.6 (25.1, 26.1)	25.9 (25.3, 26.4)	25.7 (25.2, 26.3)	26.1 (25.4, 26.9)	0.968
Sex					< 0.001
Female	61.5 (59.2, 63.8)	55.1 (52.7, 57.5)	51.5 (49.5, 53.5)	46.5 (44.3, 48.6)	
Male	38.5 (36.2, 40.8)	44.9 (42.5, 47.3)	48.5 (46.5, 50.5)	53.5 (51.4, 55.7)	
Race					< 0.001
Non-hispanic white people	48.8 (44.9, 52.7)	65.5 (62.3, 68.5)	70.9 (67.7, 73.9)	74.4 (71.2, 77.4)	
Non-hispanic black people	31.9 (28.5, 35.5)	13.7 (11.9, 15.7)	9.7 (8.3, 11.4)	8.1 (6.9, 9.5)	
Mexican American	10.0 (8.4, 11.8)	10.8 (9.1, 12.7)	9.7 (8.0, 11.7)	8.3 (6.7, 10.2)	
Other races	9.4 (7.9, 11.1)	10.1 (8.5, 11.8)	9.7 (8.2, 11.5)	9.2 (7.6, 11.2)	
Education levels					0.009
Less than 9th grade	7.0 (6.2, 8.0)	6.8 (5.9, 7.8)	5.5 (4.7, 6.4)	6.4 (5.4, 7.5)	
9–11th grade/high school grade or equivalent	39.7 (37.4, 42.0)	37.1 (34.8, 39.4)	41.6 (39.3, 43.9)	41.6 (39.0, 44.3)	
College graduate or above	53.3 (51.0, 55.5)	56.2 (53.7, 58.6)	52.9 (50.5, 55.3)	52.0 (49.4, 54.6)	
Diabetes mellitus					< 0.001
No	83.2 (81.5, 84.8)	83.4 (81.9, 84.8)	80.8 (79.2, 82.3)	76.3 (74.4, 78.2)	
Yes	16.8 (15.2, 18.5)	16.6 (15.2, 18.1)	19.2 (17.7, 20.8)	23.7 (21.8, 25.6)	
CVD					< 0.001
No	92.6 (91.4, 93.6)	92.5 (91.3, 93.6)	90.0 (88.6, 91.3)	85.9 (84.2, 87.5)	
Yes	7.4 (6.4, 8.6)	7.5 (6.4, 8.7)	10.0 (8.7, 11.4)	14.1 (12.5, 15.8)	
Hypertension					< 0.001
No	55.3 (52.9, 57.6)	54.9 (52.4, 57.5)	51.2 (48.8, 53.6)	46.7 (44.6, 48.8)	
Yes	44.7 (42.4, 47.1)	45.1 (42.5, 47.6)	48.8 (46.4, 51.2)	53.3 (51.2, 55.4)	
Hyperlipidemia					0.053
No	19.6 (17.9, 21.4)	20.7 (18.9, 22.7)	18.1 (16.3, 20.0)	17.5 (15.8, 19.3)	
Yes	80.4 (78.6, 82.1)	79.3 (77.3, 81.1)	81.9 (80.0, 83.7)	82.5 (80.7, 84.2)	
Smoking					< 0.001
Never	62.4 (60.4, 64.4)	57.4 (55.2, 59.5)	53.2 (51.0, 55.4)	48.4 (46.0, 50.8)	
Former	20.7 (18.7, 22.7)	24.9 (22.9, 26.9)	26.1 (24.2, 28.1)	28.2 (26.0, 30.5)	
Current	16.9 (15.2, 18.8)	17.8 (16.2, 19.4)	20.7 (18.8, 22.7)	23.4 (21.7, 25.2)	
Drinking					0.1
Never	14.0 (11.9, 16.4)	12.9 (11.3, 14.7)	13.4 (11.3, 15.7)	11.5 (10.0, 13.2)	
Former	19.6 (17.4, 22.0)	18.2 (16.2, 20.4)	18.8 (17.0, 20.7)	22.1 (20.1, 24.2)	
Mild/Moderate	30.4 (28.0, 33.0)	32.3 (29.7, 35.0)	32.9 (30.6, 35.2)	31.5 (29.2, 33.9)	
Heavy	36.0 (33.8, 38.3)	36.6 (34.3, 38.9)	35.0 (32.5, 37.6)	34.9 (32.6, 37.3)	
Antihypertensives					< 0.001
No	88.7 (87.2, 90.0)	88.7 (86.9, 90.3)	86.7 (85.1, 88.1)	84.3 (82.7, 85.8)	
Yes	11.3 (10.0, 12.8)	11.3 (9.7, 13.1)	13.3 (11.9, 14.9)	15.7 (14.2, 17.3)	
Glucose-lowering drugs					< 0.001
No	90.7 (89.3, 91.9)	89.4 (88.1, 90.7)	88.2 (86.8, 89.4)	85.4 (83.9, 86.8)	
Yes	9.3 (8.1,10.7)	10.6 (9.3, 11.9)	11.8 (10.6, 13.2)	14.6 (13.2, 16.1)	
Lipid-lowering drugs					< 0.001
No	86.5 (85.2, 87.8)	84.2 (82.2, 85.9)	80.2 (78.2, 82.0)	78.1 (75.9, 80.1)	
Yes	13.5 (12.2, 14.8)	15.8 (14.1, 17.8)	19.8 (18.0, 21.8)	21.9 (19.9, 24.1)	

Categorical variables were expressed as survey-weighted percentage (95% Confidence interval)

Continuous variables were expressed as survey-weighted mean (95% Confidence interval)

*ALT* Alanine aminotransferase, *AST* Aspartate aminotransferase, *TC* Total cholesterol, *HDL* High-density lipoprotein cholesterol, *CVD* cardiovascular diseases. *eGFR* estimated glomerular filtration rate

### Correlation analysis of SII, SIRI, and baseline characteristics

Spearman correlation analysis demonstrated a weak or no correlation between SII and SIRI and the baseline characteristics of the participants (all the r < 0.15). Detailed information is presented in Additional file 1: Table S2.

### SIRI, SII, and all-cause mortality

**Fig. 1:**
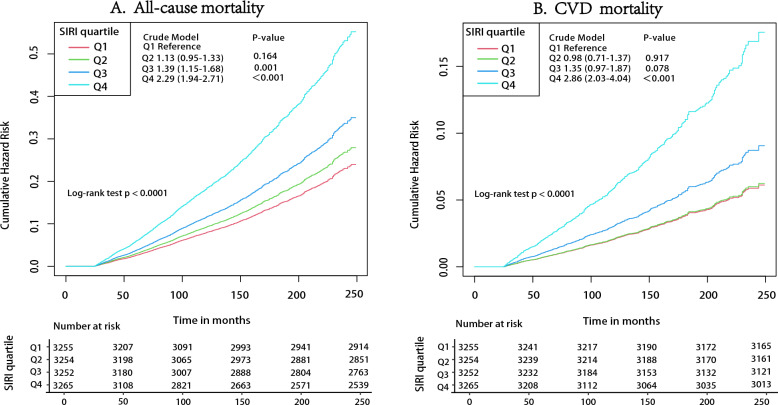
**A** Kaplan–Meier survival curves, by SIRI quartile level, for all-cause mortality. **B** Kaplan–Meier survival curves, by SIRI quartile level, for CVD mortality

During a median follow-up period of 137 months (interquartile range: 95–188 months; 150,173 person-years), there were 1959 all-cause deaths recorded. As shown in the Kaplan–Meier curves, in both the crude model (HR for SIRI in Q4 and Q1:2.29; 95% CI 1.94–2.71; P < 0.001 by log-rank test) and the multivariate-adjusted model (HR for SIRI in Q4 and Q1: 1.31; 95% CI 1.08–1.59; P < 0.05), a positive association existed between the quartile increase in SIRI and the risk of all-cause mortality (Fig. 1A; Table 2). Similarly, after full adjustment for confounders, the risk of all-cause mortality increased by 16% (HR 1.16; 95% CI 1.09–1.23; P < 0.001) as a per-SD unit increase in SIRI (Table 2). By contrast, a progressive positive association between an increase in SII quartiles and the risk of all-cause mortality in the crude model, or even in the absence of any adjusted variables, was not identified (P = 0.256) (Additional file 1: Table S3). Nevertheless, in the crude and fully adjusted models, the risk of all-cause mortality increased by 12% (HR 1.12; 95% CI 1.07–1.17) and 9% (HR 1.09; 95% CI 1.02–1.16) for each SD increase in SII, respectively.

**Table 2:** Survey-weighted cox proportional hazard results examining the association of SIRI with all-cause and cardiovascular disease mortality in the obese population. (SIRI was divided into quartiles, with the lowest group as the reference group)

	Death	Adjusted model 1	Adjusted model 2	Adjusted model 3	Adjusted model 4	Adjusted model 5	Adjusted model 6
		HR (95% CI)	HR (95% CI)	HR (95% CI)	HR (95% CI)	HR (95% CI)	HR (95% CI)
	1959	All-cause mortality					
Q1	341	Reference	Reference	Reference	Reference	Reference	Reference
Q2	403	1.06 (0.89–1.26)^c^	1.06 (0.88–1.29)^c^	0.99 (0.79–1.23)^c^	0.97 (0.78–1.19)^c^	0.97 (0.79–1.18)^c^	0.95 (0.77–1.16)^c^
Q3	489	1.22 (1.02–1.46) ^a^	1.13 (1.05–1.21)^a^	1.18 (0.93–1.49)^c^	1.10 (0.88–1.38)^c^	1.08 (0.87–1.34)^c^	1.01 (0.82–1.25)^c^
Q4	726	1.81 (1.52–2.14)^b^	2.09 (1.73–2.53)^b^	1.66 (1.35–2.04)^b^	1.47 (1.20–1.81)^b^	1.43 (1.18–1.75)^b^	1.30 (1.07–1.58)^a^
P for trend		< 0.001	< 0.001	< 0.001	< 0.001	< 0.001	< 0.001
Per SD increase		1.23 (1.16–1.31)^b^	1.27 (1.22–1.34)^b^	1.20 (1.15–1.26)^b^	1.18 (1.13–1.24)^b^	1.18 (1.11–1.25)^b^	1.16 (1.09–1.23)^b^
	553	Cardiovascular disease mortality					
Q1	90	Reference	Reference	Reference	Reference	Reference	Reference
Q2	93	0.91 (0.66–1.27)^c^	0.89 (0.62–1.29)^c^	0.88 (0.58–1.34)^c^	0.83 (0.55–1.27)^c^	0.83 (0.55–1.26)^c^	0.87 (0.57–1.33)^c^
Q3	131	1.17 (0.86–1.58)^c^	1.14 (0.81–1.59)^c^	1.09 (0.73–1.62)^c^	0.98 (0.66–1.48)^c^	0.96 (0.64–1.42)^c^	0.88 (0.59–1.30)^c^
Q4	239	2.16 (1.55–3.01)^b^	2.02 (1.41–2.88)^b^	1.99 (1.32–2.99)^a^	1.70 (1.12–2.59)^a^	1.62 (1.08–2.42)^a^	1.57 (1.05–2.37)^a^
P for trend		< 0.001	< 0.001	< 0.001	< 0.001	0.002	0.004
Per SD increase		1.28 (1.18–1.39)^b^	1.33 (1.24–1.41)^b^	1.25 (1.16–1.34)^b^	1.24 (1.14–1.34)^b^	1.22 (1.11–1.35)^b^	1.22 (1.10–1.36)^b^

Model 1 adjust age

Model 2 adjust Model 1 plus other demographic variables including sex, race, education levels and poverty income ratio

Model 3 adjusted Model 2 plus other parameters including body mass index, estimated glomerular filtration rate, alanine aminotransferase, total cholesterol, and high-density lipoprotein cholesterol

Model 4 adjusted Model 3 plus history of diseases including cardiovascular diseases, diabetes mellitus, hypertension, and hyperlipidemia

Model 5 adjusted Model 4 plus medication including antihypertensives, glucose-lowering and lipid-lowering drugs

Model 6 adjusted Model 5 plus lifestyle variables including smoking and drinking

^a^Indicates p-value < 0.05

^b^Indicates p-value < 0.001

^c^Indicates p-value ≥ 0.05

**Fig. 2:**
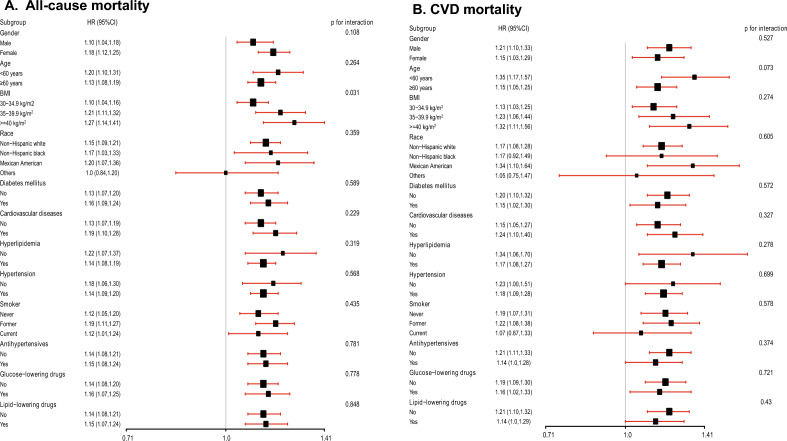
**A** Subgroup analyses of the associations (hazard ratios, 95% CIs) between SIRI increase and the risk of all-cause mortality. HRs indicate the increased risk of all-cause mortality for each standard deviation increase in SIRI. **B** Subgroup analyses of the associations (hazard ratios, 95% CIs) between SIRI increase and the risk of CVD mortality. HRs indicate the increased risk of all-cause mortality for each standard deviation increase in SIRI. HR has been fully adjusted by age, sex, race, education levels and poverty income ratio, body mass index, estimated glomerular filtration rate, alanine aminotransferase, total cholesterol, high-density lipoprotein cholesterol, cardiovascular diseases, diabetes mellitus, hypertension, hyperlipidemia, antihypertensives, glucose-lowering drugs, lipid-lowering drugs, smoking, and drinking

As shown in the subgroup analysis, the positive association between SIRI and risk of all-cause mortality was consistent across all strata except the BMI group (all P-values for interaction > 0.05), showing a significant positive trend between SIRI and all-cause mortality in most groups. Except for other races, the risk of all-cause mortality increased by 10–26% with each SD increase in SIRI (Fig. 2A). However, an interaction (p-value for interaction = 0.034) was observed in the BMI group. Notably, the relationship between SIRI and the risk of all-cause mortality increased significantly with increasing BMI. Similarly, the positive association between SII and all-cause mortality risk was consistent across all strata except for the diabetes mellitus and glucose-lowering drug groups (all P values for interactions > 0.05). Across most strata, the risk of all-cause mortality increased from 5 to 20% per SD increase in SII (Additional file 1: Figure S2A).

### SIRI, SII, and CVD mortality

A total of 553 CVD deaths were observed during follow-up. Consistent with the results for all-cause mortality in the crude model (HR for SIRI in the Q4 and Q1: 2.86; 95% CI 2.03–4.04; P < 0.001 by log-rank test) and in the multivariate-adjusted model (HR for SIRI in the fourth quartile and first quartile: 1. 57; 95% CI 1.05–2.37; P < 0.05), a positive association existed between increased quartiles of SIRI and risk of CVD mortality in both models (Fig. 1B; Table 2). Similarly, after full adjustment for confounders, a per-SD increase in SIRI was related to a 22% increase in the risk of CVD mortality (HR 1.22; 95% CI 1.10–1.36; P < 0.001) (Table 2). On the contrary, a positive association between quartile increase in SII and risk of all-cause mortality in the crude model was not found (P = 0.104) (Additional file 1: Table S3). Nevertheless, in the crude and fully adjusted models, the risk of CVD mortality increased by 15% (HR 1.15; 95% CI 1.06–1.24) and 14% (HR 1.14; 95% CI 1.04–1.26) with every SD unit increase in SII, respectively.

In the subgroup analysis, the association between SIRI and CVD mortality risk was consistent across all strata (all P values for interactions > 0.05). Except for Non-Hispanic black and other races, the risk of CVD mortality increased by 15–35% with a per-SD increase in SIRI (Fig. 2B), which implies that higher SIRI was positively associated with the risk of all-cause mortality in most subgroups. Likewise, the relation between SII and risk of CVD mortality was consistent across all strata (all P values for interactions > 0.05). In most strata, the risk of CVD mortality increased from 9 to 38% for each SD increase in SII (Additional file 1: Figure S2B).

### Dose-dependent relationship

**Fig. 3:**
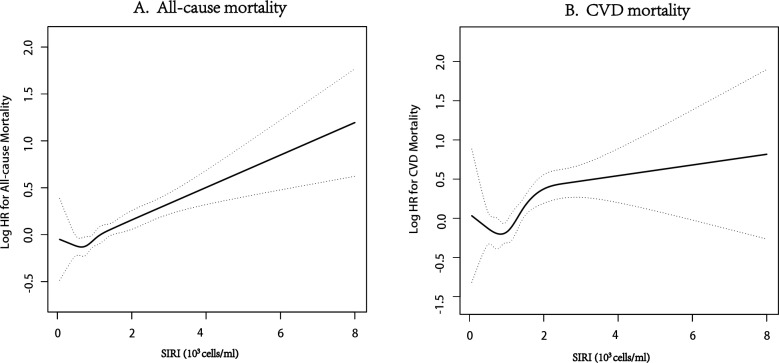
**A** The smooth curve of the relationship between SIRI levels and the risk of all-cause mortality.** B** The smooth curve of the relationship between SIRI levels and the risk of CVD mortality. HR has been fully adjusted by age, sex, race, education levels and poverty income ratio, body mass index, estimated glomerular filtration rate, alanine aminotransferase, total cholesterol, high-density lipoprotein cholesterol, cardiovascular diseases, diabetes mellitus, hypertension, hyperlipidemia, antihypertensives, glucose-lowering drugs, lipid-lowering drugs, smoking, and drinking

As presented in Fig. 3A, SIRI levels were linearly related to the risk of all-cause mortality (P for nonlinear = 0.492). Regarding each 1-unit increase in SIRI, the risk of all-cause mortality increased by 20% (HR 1.20; 95% CI 1.13–1.28; P < 0.001). However, SIRI levels were nonlinearly associated with the risk of CVD mortality (P for nonlinear = 0.039), as displayed in Fig. 3B. Threshold effect analysis exhibited a 50% increase in the risk of CVD mortality with a 1-unit increase in SIRI when SIRI < 2.27 (HR 1.50; 95% CI 1.23–1.82; P < 0.001). However, no significant association existed between SIRI and the risk of CVD mortality when SIRI ≥ 2.27 (HR 1.05; 95% CI 0.85–1.31; P = 0.642) (Additional file 1: Table S4). In addition, since P for nonlinearity approached 0.05, the linear relationship between SIRI and the risk of CVD mortality was also reported. Furthermore, a 1-unit increase in SIRI was associated with a 26% (HR 1.26; 95% CI 1.14–1.40; P < 0.001) increase in the risk of CVD mortality.

Similarly, SII levels were linearly related to the risk of all-cause mortality (P for nonlinear = 0.152) (Additional file 1: Figure S3A, Table S4). Precisely, a 100-unit increase in SII corresponded to a 3% increase in the risk of all-cause mortality (HR 1.03; 95% CI 1.02–1.05; P < 0.001) (Additional file 1: Table S4). By contrast, SII levels demonstrated a nonlinear relationship with the risk of CVD mortality (P for nonlinear = 0.027) (Additional file 1: Figure S3B). Threshold effect analysis revealed that a 100-unit increase in SII corresponded to a 126% increase in the risk of CVD mortality when SII was < 246 (HR 2.26; 95% CI 1.04–4.88; P = 0.04). However, a 100-unit increase in SII was correlated with only a 4% increase in the risk of CVD mortality when SII was ≥ 246 (HR 1.04; 95% CI 1.02–1.07; P = 0.002) (Additional file 1: Table S4). In addition, the linear relationship between SII and the risk of CVD mortality was also observed when the nonlinear P-value was close to 0.05. A 100-unit increase in SII corresponded to a 5% increase in the risk of CVD mortality (HR 1.05; 95% CI 1.02–1.08; P < 0.001) (Additional file 1: Table S4).

### Construction of the clinical model with 10-year survival probability

**Fig. 4:**
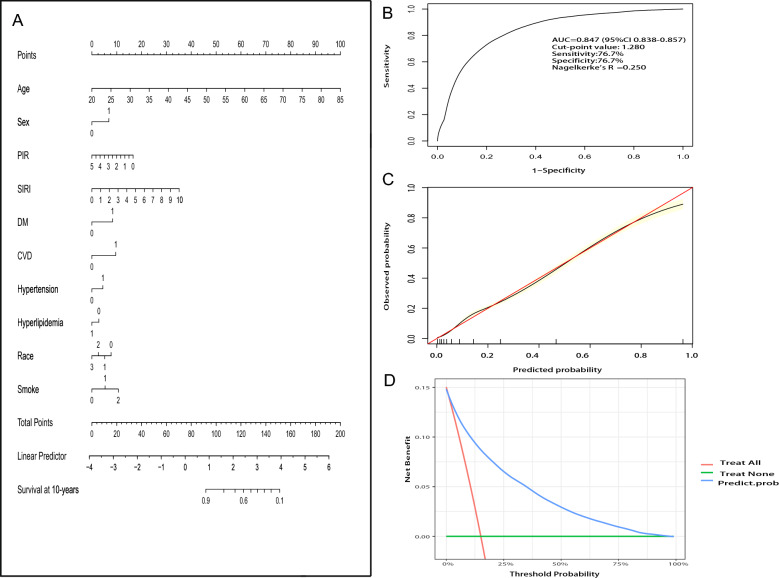
**A** Nomogram for predicting the probability of 10-year survival. The top horizontal line is the score column, where the total score summed item by item corresponds to the corresponding 10-year probability of survival according to the score of each item. In the sex groups, 0 means “women,” and 1 means “men.” In the CVD, DM, hypertension, and hyperlipidemia groups, 0 means “no,” and 1 means “yes.” In the smoking group, 0 indicates “never,” 1 indicates “former,” and 2 indicates “current.” In the race group, 0 indicates “non-Hispanic white people,” 1 indicates “non-Hispanic black people,” 2 indicates “Mexican American,” and 3 indicates “other races.” **B** Nomogram predicting the ROC curve for 10-year survival in the obese population. **C** Calibration curves predicting 10-year survival in the obese population. The x-axis is the predicted probability, and the y-axis is the observed probability. The red line is the ideal prediction model, where the predicted outcome is consistent with the observed outcome. The black line is the predicted outcome of the nomogram. **D** Decision curve analysis describing the net clinical benefit. Green line = net benefit when all participants are not intervened. Redline = net benefit where all participants will be subjected to the intervention. Blue line = predicted outcome of the nomogram

As depicted in Additional file 1: Figure S4, the predictive value of SIRI (AUC = 0.601 and 0.624) for all-cause and CVD mortality surpassed that of SII (AUC = 0.528 and 0.539). As a result, the SIRI model was selected for constructing the clinical model. Through plotting nomograms, the results of the clinical model were visualized. Baseline characteristics were screened by univariate analysis of variance and multivariate Cox regression (Additional file 1: Table S5, S6). Finally, 10 variables (age, sex, race, PIR, SIRI, DM, CVD, hypertension, hyperlipidemia, and smoke) were screened to construct a nomogram to predict 10-year survival probability (Fig. 4A). The model showed good discriminatory power with an AUC of 0.847 (95% CI 0.838–0.857), and Nagelkerke's R^2^ was 0.250 *(*Fig. 4B). Meanwhile, the calibration curve indicated that the nomogram had a similar performance to the ideal model, implying that the nomogram had better predictive power. However, the prediction risk of the model may be high when the risk is greater than 80% (Fig. 4C). As presented in the decision curve, the threshold probability is within 0% to 100%, and a net benefit can be obtained using this nomogram, suggesting that the model has a promising clinical application prospect (Fig. 4D).

## Discussion

This study is the first attempt to investigate the association between SIRI and SII levels and all-cause and CVD mortality in the obese population. The main findings of this study are worth noting. Firstly, both SIRI and SII are stable inflammatory markers showing a negligible correlation with baseline clinical characteristics. Secondly, in the obese population, SIRI and SII are independent risk factors for all-cause and CVD mortality, with a linear association for all-cause mortality and a nonlinear association for CVD mortality. Thirdly, by comparing their predictive value for all-cause and CVD mortality, we established that SIRI exhibited a superior prognostic value relative to SII for the first time. Furthermore, a robust clinical prediction model incorporating SIRI for estimating the 10-year risk of mortality with significant sensitivity and specificity exceeding 75% was developed, and its high predictive value was also validated by the decision curve, revealing its clinically solid applicability for risk assessment.

SIRI and SII were initially proposed as prognostic indicators for cancer patients' adverse outcomes due to their ability to reflect the systemic inflammatory response [30, 38]. Our study established both SIRI and SII as stable systemic inflammatory markers showing negligible associations with baseline clinical characteristics. These systemic inflammatory indices are practical for clinical use, as they are easily obtained by counting blood cells. Inflammation plays a crucial role in tumorigenesis and the development of CVD [20, 39]. Elevated SIRI levels indicated a positive correlation with the severity of coronary artery disease (CAD) (including three-vessel CAD and acute coronary syndrome) and an increased incidence of myocardial infarction (MI) [40, 41]. Zhao et al. revealed that each 1-unit increase in continuous SIRI is correlated with a 30% higher hazard of all-cause and CVD mortality in hypertensive patients [25]. Besides, Cao et al. demonstrated that higher SII shows a significant relationship to an increased risk of cardiovascular disease (CVD) mortality in hypertensive patients, and there is a nonlinear association between SII and all-cause mortality [27].

Meanwhile, Xiao et al. found a nonlinear correlation between the SII index and all-cause and CVD mortality in patients with CVD [42]. Our results suggest that elevated levels of SIRI and SII are associated with increased CVD and all-cause mortality risk to a great extent. Specifically, increasing quartiles of SIRI is related to a 31% higher risk of all-cause mortality and a 57% higher risk of CVD mortality. A per-SD increase in SIRI is connected with a 16% and 23% higher risk of all-cause and CVD mortality, respectively. By contrast, increasing quartiles of SII does not dramatically increase the risk of all-cause and CVD mortality, while each SD unit increase in SII is associated with a 9% and 14% higher risk of all-cause and CVD mortality, respectively. Thus, SIRI may confer a greater risk of all-cause and CVD mortality for the obese population than SII, even though both are independent risk factors.

In addition, other studies have also shown that SIRI and SII exert distinct effects on cardiovascular diseases. For example, Jin et al. discovered that elevated SIRI, but not SII, was associated with increased myocardial infarction (MI) in the general population. In contrast, elevated SIRI and SII correlated with an increased risk of stroke and all-cause death [40]. Dziedzic et al. found that SIRI, but not SII, was positively correlated with the severity of coronary artery disease (CAD) [41]. Moreover, our study provides novel insight into the distinct predictive capabilities of SIRI and SII in evaluating mortality risk through direct comparison, effectively addressing the current gap in knowledge within this field. The predictive value of SIRI for all-cause and CVD mortality was found to surpass that of SII, implying that SIRI represents a more valuable marker for predicting all-cause and CVD mortality for the obese population. The variance in SIRI and SII cellular composition may explain the differential impact on cardiovascular diseases. Even though both SIRI and SII encompass neutrophil and lymphocyte counts, their discriminative attribute resides in including additional cell types for analysis. Specifically, SIRI considers monocyte counts, while SII focuses on platelet counts. Monocytes play a vital role in the pathogenesis of atherosclerosis by directly contributing to various essential processes, such as foam cell formation, secretion of cytokines and chemokines, and destabilization of plaques [43–45]. An increased monocyte count is correlated with a heightened risk of cardiovascular disease (CVD) mortality, independent of conventional risk factors [46–48].

Moreover, in the context of obesity, elevated leptin levels promote the attraction of monocytes and macrophages to blood vessels, producing inflammatory and adhesion molecules [49–51]. Leptin also stimulates the production of reactive oxygen species and facilitates the chemoattraction and proliferation of monocytes/macrophages in endothelial cells [52]. These effects cause endothelial dysfunction and atherosclerosis by impairing nitric oxide-dependent vasorelaxation and inducing vasoconstriction and thrombosis [53, 54]. Conversely, elevated platelet counts are not directly linked to cardiovascular events, whereas activated and aggregated platelets can cause arterial clot formation, obstructing blood flow and causing heart attacks or strokes [55–59]. Therefore, the primary therapeutic strategy for coronary heart disease or ischemic strokes primarily revolves around preventing platelet activation and aggregation rather than lowering platelet counts [60, 61]. Considering the role of monocyte and platelet counts in atherosclerosis and its pathogenesis, it is reasonable to assume that among obese individuals, elevated monocyte counts have a more significant impact on CVD mortality compared with elevated platelet counts.

In addition, the impact of the remaining SIRI and SII cell types on systemic inflammation and the development of atherosclerosis needs to be further considered. Specifically, neutrophils function critically in the inflammatory response associated with atherosclerosis, as they release various inflammation mediators, chemotactic agents, and reactive oxygen species that can lead to endothelial cell damage and tissue ischemia [62–64]. On the contrary, lymphocytes exhibit a regulatory function in inflammation and may exert an inhibitory effect on atherosclerosis [65–68]. Numerous studies have consistently supported the abovementioned findings, demonstrating a positive correlation between elevated levels of monocytes and neutrophils and decreased levels of lymphocytes with an increased risk of cardiovascular disease [69, 70]. Consistent with the obtained observations, it was observed that individuals with higher levels of SIRI were characterized by elevated levels of neutrophils and monocytes while exhibiting lower levels of lymphocytes. Notably, a robust clinical prediction model incorporating SIRI, enabling the estimation of 10-year mortality risk with significant sensitivity and specificity exceeding 75%, was developed. SIRI, which involves the counts of monocytes, neutrophils, and lymphocytes, is a valuable tool for accurately predicting mortality risk in individuals with obesity. Moreover, the developed clinical prediction model provides a practical approach for implementing SIRI in clinical practice.

Currently, the use of SIRI and SII in clinical prognosis remains limited. A few studies have shown that SIRI and SII can reliably predict the efficacy of anti-inflammatory therapies in treating specific inflammation-related disorders such as chronic spontaneous urticaria and acne vulgaris [71, 72]. However, broader investigations are necessary to validate the potential roles of these novel inflammatory markers in the prognosis of conditions like obesity and CVD.

Recently, there has been increased attention to anti-inflammatory therapy for CVD. Several studies have used hs-CRP, a traditional inflammation indicator, as a metric to assess anti-inflammatory efficacy. Canakinumab has been shown to significantly reduce the rate of recurrent cardiovascular events in patients with a history of myocardial infarction. However, canakinumab did not affect all-cause and CVD mortality but was associated with a higher incidence of fatal infections [73]. In contrast, low-dose colchicine reduced all-cause and cardiovascular disease mortality but was not associated with a significant increase in fatal infections [74]. Additionally, many studies have shown that using SIRI or SII provides significant advantages over hs-CRP in assessing therapeutic efficacy and risk stratification for CVD patients [75–79]. Therefore, SIRI and SII are likely to become key tools for assessing inflammation levels in obese individuals and gauging the effectiveness of anti-inflammatory interventions.

The global issue of obesity poses a significant obstacle to promoting lifelong health and preventing chronic diseases; however, the criteria and methods currently used to diagnose obesity remain controversial [80]. A systematic review and meta-analysis determined that the BMI ≥ 30 kg/m^2^ for diagnosing obesity exhibits a notable specificity of 0.97 (CI 0.96–0.97) but a limited sensitivity of 0.42 (CI 0.31–0.43), indicating that individuals with a BMI ≥ 30 kg/m2 are less likely to encounter misclassification as non-obese [81]. It is recognized that the health risks associated with excess weight in specific populations are usually evident at lower BMI levels [82, 83]. The guidelines recommend annual BMI screening for all adults to assess overweight and obesity [84, 85]. For most people, a BMI of 25 kg/m^2^ warrants further assessment; for individuals of Asian descent, however, an assessment may be required at a BMI of 23 kg/m^2^. Current BMI criteria lead to the underdiagnosis of excess adiposity in normal-weight individuals; therefore, BMI is not a perfect measure of excessive or abnormal accumulation of body fat. The BMI cut-off of 30 kg/m^2^ universally identifies obesity and offers significant specificity. As such, this cut-off value continues to be used as a benchmark for statistical identification and classification of obesity in reports published by the WHO, the CDC, and the NIH, as well as in many studies [1, 2, 86, 87]. This study also used this criterion to identify obesity, with a particular emphasis on the prognostic value of SIRI and SII on all-cause and CVD mortality in this population.

The primary demographic composition of this study included non-Hispanic whites, non-Hispanic blacks, and Mexican Americans. The multiracial group, comprising less than 10%, included Asians as well as other minorities. Within the multiracial group, a higher proportion of individuals were classified as Class I obese; their mean BMI was slightly lower compared to the predominant races (as depicted in Additional file 1: Figures S5, S6). Given the limited representation of Asian populations, obese individuals with lower BMIs might not have necessarily presented a higher risk. In our subgroup analyses, we observed that elevated SIRI and SII were associated with increased all-cause and CVD mortality across each subgroup categorized by BMI. Notably, an interaction effect was observed among the BMI subgroups, indicating that higher SIRI and BMI levels influenced the elevated all-cause mortality. Therefore, we further evaluated the predictive efficacy of SIRI compared to BMI for mortality. The results indicated that the predictive value of SIRI for both all-cause and CVD mortality surpassed that of BMI (as depicted in Additional file 1: Figure S7), suggesting SIRI was a superior predictor for the obese population. In addition, when race, BMI, and other potential confounders were fully adjusted for in the regression models, the results consistently showed that SIRI and SII were independent risk factors for all-cause mortality and cardiovascular mortality. Thus, neither BMI levels nor race influenced the study’s predictive validity.

### Limitations

However, this study still has several limitations. First, as an observational study, a causal relationship between SIRI and all-cause or cardiovascular mortality cannot be established. Second, as the study population was limited to Americans, the generalizability of our findings to other populations may be limited. Thirdly, our study's small number of participants with SIRI > 2.5 may have influenced the observed nonlinear association between SIRI and CVD mortality. Third, missing data on other inflammatory markers, including C-reactive protein level, could not be included in our analysis of baseline characteristics, potentially influencing the results. Finally, the United States is a multiethnic country. The demographic composition of the present study consisted primarily of non-Hispanic whites, non-Hispanic blacks, and Mexican Americans. Multiracial populations, including Asians and other minorities, make up less than 10% of the total population. Therefore, the cutoff value for obesity might vary because of differences in body composition and health risks among different races. In our study, we identified the obese population based on a BMI ≥ 30 kg/m^2^.While this is a generally accepted standard, it might not be fully applicable to all racial and ethnic groups, particularly Asians.

## Conclusion

To conclude, SIRI and SII are independent risk factors for all-cause and CVD mortality in the obese population. The ability of SIRI to predict all-cause and CVD mortality significantly outperforms that of SII, suggesting that SIRI is a more valuable marker of inflammation. Moreover, the construction of predictive models has augmented the clinical utility of SIRI. Using prognostic nomograms, clinicians can identify obese individuals at an elevated risk of death and devise suitable interventions to improve their health outcomes.

### Supplementary Information

**Additional file 1: Figure S1.** The flow of the study chart. **Figure S2.** A Subgroup analyses of the associations (hazard ratios, 95% CIs) between SII increase and the risk of all-cause mortality. HRs indicate the increased risk of all-cause mortality for each standard deviation increase in SII. B Subgroup analyses of the associations (hazard ratios, 95% CIs) between SII increase and the risk of CVD mortality. **Figure S3.** A Smooth curve of the relationship between SII levels and the risk of all-cause mortality. B Smooth curve of the relationship between SII levels and the risk of CVD mortality. **Figure S4.** A Comparison of SIRI and SII predictions of all-cause mortality. B Comparison of SIRI and SII predictions of CVD mortality. **Figure S5.** Comparison of BMI levels between the major races and other multiracial groups. **Figure S6.** Comparison of proportions of major races and other multiracial groups under different BMI categories. **Figure S7.** Comparisons of SIRI and SII versus BMI for prediction of all-cause and CVD mortality. **Table S1.** Survey-weighted baseline characteristics of the obese population in NHANES from 1999 to 2014 according to SII quartiles (N=13,026, representing 63,479,085 individuals with obesity). **Table S2.** Spearman correlation analysis of SIRI, SII, and baseline characteristics. **Table S3.** Survey-weighted cox proportional hazard results examining the association of SII with all-cause and cardiovascular disease mortality in the obese population. (SII was divided into quartiles, with the lowest group as the reference group). **Table S4.** Threshold-effect analysis on SIRI, SII, and all-cause and CVD mortality. **Table S5.** Differences in the baseline characteristics of survivors and the death. **Table S6.** Multivariate cox regression to assess the relationship between baseline variables and all-cause mortality.

## Data Availability

Data are publicly available at https://www.cdc.gov/nchs/nhanes/index.htm.
