# Supplementary material for: System inflammation response index: a novel inflammatory indicator to predict all-cause and cardiovascular disease mortality in the obese population
Source: Diabetol Metab Syndr. 2023 Oct 11;15:195. doi: 10.1186/s13098-023-01178-8 (PMC10566161; doi:10.1186/s13098-023-01178-8)

| **Table S1. Survey-weighted baseline characteristics of the obese population in NHANES from 1999 to 2014 according to SII quartiles (N=13,026, representing 63,479,085 individuals with obesity).** | | | | | |
| --- | --- | --- | --- | --- | --- |
|  | Q1  (<358.1) | Q2  (358.1-504) | Q3  (504-699.4) | Q4  (<699.4) | P-value |
| **Participants, n** | 3255 | 3254 | 3252 | 3265 |  |
| **Platelets (×10^3^ cells/ml)** | 218.5 (216.1, 220.9) | 249.3 (247, 251.5) | 272.9 (269.8, 275.9) | 315.7 (312.2, 319.2) | <0.001 |
| **Neutrophils (×10^3^ cells/ml)** | 3.2 (3.1, 3.3) | 4.1 (4.0, 4.2) | 4.7 (4.6, 4.8) | 6.1 (6.0, 6.2) | <0.001 |
| **Lymphocytes (×10^3^cells/ml)** | 2.6 (2.5, 2.7) | 2.3 (2.2,2.4) | 2.2 (2.1, 2.3) | 2.0 (1.9. 2.1) | <0.001 |
| **Age (years)** | 47.1 (46.3, 47.9) | 46.4 (45.7, 47.2) | 45.8 (45.1, 46.4) | 45.7 (45.0, 46.5) | 0.006 |
| **Poverty income ratio** | 2.7 (2.7 ,2.8) | 2.9 (2.8 ,3.0) | 2.9 (2.8 ,3.0) | 2.8 (2.7, 2.9) | 0.002 |
| **Body mass index (Kg/m^2^)** | 35.1 (34.9, 35.3) | 35.3 (35.0, 35.5) | 35.7 (35.4, 35.9) | 36.8 (36.6, 37.1) | <0.001 |
| **HDL** (mmol/L) | 1.2 (1.2, 1.2) | 1.2 (1.2, 1.2) | 1.2 (1.2, 1.2) | 1.2 (1.2, 1.2) | 0.032 |
| **TC** (mmol/L) | 5.1 (5.1, 5.2) | 5.1 (5.1, 5.2) | 5.2 (5.1, 5.2) | 5.1 (5.1, 5.2) | 0.151 |
| **eGFR** | 95.2 (94.2, 96.3) | 95.2 (94.1, 96.3) | 95.2 (94.3, 96.2) | 95.0 (94.0, 96.1) | 0.982 |
| **ALT (U/L)** | 30.9 (29.9, 31.9) | 29.8 (28.9, 30.7) | 28.8 (27.8, 29.7) | 26.8 (26.0, 27.6) | <0.001 |
| **AST (U/L)** | 27.5 (26.9, 28.2) | 26.0 (25.5, 26.5) | 25.7 (24.9, 26.4) | 24.5 (23.8, 25.1) | <0.001 |
| **Sex** |  |  |  |  | <0.001 |
| Female | 45.8 (43.9, 47.7) | 49.3 (46.9, 51.7) | 52.9 (50.7, 55.0) | 63.2 (60.9, 65.3) |  |
| Male | 54.2 (52.3, 56.1) | 50.7 (48.3, 53.1) | 47.1 (45.0, 49.3) | 36.8 (34.7, 39.1) |  |
| **Race** |  |  |  |  | <0.001 |
| Non-Hispanic white people | 55.9 (51.8, 59.9) | 66.0 (62.9, 69.1) | 69.0 (65.9, 72.0) | 70.5 (67.0, 73.7) |  |
| Non-Hispanic black people | 25.5 (22.3, 28.9) | 14.6 (12.8, 16.5) | 11.3 (9.8, 13.0) | 10.3 (8.9, 12.0) |  |
| Mexican American | 10.1 (8.5, 11.9) | 9.7 (8.0, 11.6) | 9.6 (8.1, 11.4) | 9.3 (7.6, 11.5) |  |
| Other races | 8.6 (7.2, 10.4) | 9.7 (8.2, 11.5) | 10.0 (8.4, 11.9) | 9.8 (8.2, 11.8) |  |
| **Education levels** |  |  |  |  | 0.013 |
| Less than 9th grade | 7.6 (6.7, 8.7) | 6.4 (5.5, 7.5) | 5.9 (5.1, 6.9) | 5.7 (4.9, 6.7) |  |
| 9-11th grade/high school grade or equivalent | 41.2 (38.9, 43.6) | 37.9 (35.9, 40.0) | 39.7 (37.1, 42.4) | 41.4 (39.0, 43.9) |  |
| College graduate or above | 51.1 (48.8, 53.5) | 55.6 (53.4, 57.8) | 54.3 (51.5, 57.2) | 52.8 (50.4, 55.3) |  |
| **Diabetes mellitus** |  |  |  |  | 0.052 |
| No | 80.8 (79.2, 82.2) | 81.1 (79.2, 82.9) | 82.1 (80.6, 83.5) | 79.1 (77.3, 80.7) |  |
| Yes | 19.2 (17.8, 20.8) | 18.9 (17.1, 20.8) | 17.9 (16.5, 19.4) | 20.9 (19.3, 22.7) |  |
| **CVD** |  |  |  |  | 0.117 |
| No | 89.5 (88.2, 90.8) | 90.2 (88.6, 91.7) | 91.2 (90.1, 92.2) | 89.2 (87.9, 90.4) |  |
| Yes | 10.5 (9.2, 11.8) | 9.8 (8.3, 11.4) | 8.8 (7.8, 9.9) | 10.8 (9.6, 12.1) |  |
| **Hypertension** |  |  |  |  | 0.792 |
| No | 51.8 (49.5, 54.1) | 52.2 (49.8, 54.6) | 52.2 (50.1, 54.3) | 50.9 (48.7, 53.1) |  |
| Yes | 48.2 (45.9, 50.5) | 47.8 (45.4, 50.2) | 47.8 (45.7, 49.9) | 49.1 (46.9, 51.3) |  |
| **Hyperlipidemia** |  |  |  |  | 0.017 |
| No | 21.4 (19.7, 23.3) | 19.0 (17.2, 21.0) | 17.7 (16.0, 19.4) | 18.0 (16.3, 19.8) |  |
| Yes | 78.6 (76.7, 80.3) | 81.0 (79.0, 82.8) | 82.3 (80.6, 84.0) | 82.0 (80.2, 83.7) |  |
| **Smoking** |  |  |  |  | 0.272 |
| Never | 54.9 (52.3, 57.4) | 56.2 (53.9, 58.4) | 56.0 (53.7, 58.3) | 52.7 (50.2, 55.1) |  |
| Former | 25.3 (23.0, 27.8) | 25.1 (23.4, 26.8) | 24.3 (22.4, 26.2) | 26.1 (24.1, 28.2) |  |
| Current | 19.8 (18.0, 21.7) | 18.8 (17.1, 20.5) | 19.7 (18.0, 21.6) | 21.2 (19.5, 23.1) |  |
| **Drinking** |  |  |  |  | 0.731 |
| Never | 12.2 (10.7, 13.9) | 13.1 (11.0, 15.5) | 13.0 (11.2, 15.1) | 13.1 (11.5, 14.9) |  |
| Former | 19.6 (17.7, 21.5) | 19.4 (17.5, 21.5) | 19.4 (17.7, 21.3) | 20.4 (18.7, 22.3) |  |
| Mild/Moderate | 31.5 (29.3, 33.8) | 32.6 (30.2, 35.1) | 33.0 (30.5, 35.5) | 30.3 (28.2, 32.4) |  |
| Heavy | 36.7 (34.5, 39.0) | 34.9 (32.7, 37.1) | 34.6 (32.4, 36.9) | 36.2 (34.0, 38.4) |  |
| **Antihypertensives** |  |  |  |  | 0.16 |
| No | 87.8 (86.2, 89.2) | 87.8 (86.2, 89.3) | 86.5 (85.0, 88.0) | 85.9 (84.4, 87.3) |  |
| Yes | 12.2 (10.8, 13.8) | 12.2 (10.7, 13.8) | 13.5 (12.0, 15.0) | 14.1 (12.7, 15.6) |  |
| **Glucose-lowering drugs** |  |  |  |  | 0.188 |
| No | 88.4 (87.0, 89.6) | 88.4 (86.8, 89.9) | 89.2 (87.9, 90.4) | 87.2 (85.8, 88.5) |  |
| Yes | 11.6 (10.4, 13.0) | 11.6 (10.1, 13.2) | 10.8 (9.6, 12.1) | 12.8 (11.5, 14.2) |  |
| **Lipid-lowering drugs** |  |  |  |  | 0.254 |
| No | 82.4 (80.7, 83.9) | 81.1 (78.8, 83.2) | 83.1 (81.5, 84.7) | 81.3 (79.6, 82.9) |  |
| Yes | 17.6 (16.1, 19.3) | 18.9 (16.8, 21.2) | 16.9 (15.3, 18.5) | 18.7 (17.1, 20.4) |  |

**Categorical variables were expressed as survey-weighted percentage (95% Confidence interval).**

**Continuous variables were expressed as survey-weighted mean (95% Confidence interval).**

ALT, Alanine aminotransferase. AST, Aspartate aminotransferase.TC, Total cholesterol. HDL, High-density lipoprotein cholesterol. CVD, cardiovascular diseases. eGFR, estimated glomerular filtration rate.

| **Table S2. Spearman correlation analysis of SIRI, SII, and baseline characteristics** | | | | | | | |
| --- | --- | --- | --- | --- | --- | --- | --- |
|  | **Correlation(r)** | **P-value** | **Characteristics** |  | **Correlation(r)** | **P-value** | Method |
| **SIRI** | 0.0581 | 0.0000 | **Age** | **SII** | -0.0564 | 0.0000 | spearman |
| **SIRI** | 0.1187 | 0.0000 | **Sex** | **SII** | -0.1157 | 0.0000 | spearman |
| **SIRI** | -0.1313 | 0.0000 | **Race** | **SII** | -0.0586 | 0.0000 | spearman |
| **SIRI** | -0.0043 | 0.6298 | **Education levels** | **SII** | 0.0209 | 0.0201 | spearman |
| **SIRI** | 0.0108 | 0.2378 | **PIR** | **SII** | -0.0047 | 0.6044 | spearman |
| **SIRI** | 0.0465 | 0.0000 | **BMI** | **SII** | 0.0919 | 0.0000 | spearman |
| **SIRI** | -0.0651 | 0.0000 | **TC** | **SII** | 0.0002 | 0.9835 | spearman |
| **SIRI** | -0.0964 | 0.0000 | **HDL** | **SII** | 0.0013 | 0.8851 | spearman |
| **SIRI** | 0.0210 | 0.0175 | **ALT** | **SII** | -0.0906 | 0.0000 | spearman |
| **SIRI** | -0.0116 | 0.1913 | **AST** | **SII** | -0.1374 | 0.0000 | spearman |
| **SIRI** | -0.1070 | 0.0000 | **eGFR** | **SII** | 0.0160 | 0.0710 | spearman |
| **SIRI** | 0.1069 | 0.0000 | **CVD** | **SII** | 0.0172 | 0.0551 | spearman |
| **SIRI** | 0.0576 | 0.0000 | **DM** | **SII** | 0.0055 | 0.5311 | spearman |
| **SIRI** | 0.0268 | 0.0022 | **Hyperlipidemia** | **SII** | 0.0290 | 0.0009 | spearman |
| **SIRI** | 0.0611 | 0.0000 | **Hypertension** | **SII** | -0.0079 | 0.3661 | spearman |
| **SIRI** | 0.1000 | 0.0000 | **Smoking** | **SII** | 0.0228 | 0.0111 | spearman |
| **SIRI** | 0.0063 | 0.5015 | **Alcohol use** | **SII** | 0.0008 | 0.9308 | spearman |
| **SIRI** | 0.0493 | 0.0000 | **Antihypertensives** | **SII** | 0.0160 | 0.0680 | spearman |
| **SIRI** | 0.0450 | 0.0000 | **Glucose-lowering drugs** | **SII** | 0.0051 | 0.5632 | spearman |
| **SIRI** | 0.0745 | 0.0000 | **Lipid-lowering drugs** | **SII** | -0.0074 | 0.3995 | spearman |

PIR, Poverty income ratio. BMI, Body mass index. ALT, Alanine aminotransferase. AST, Aspartate aminotransferase.TC, Total cholesterol. HDL, High-density lipoprotein cholesterol. eGFR, estimated glomerular filtration rate. DM, diabetes mellitus. CVD, cardiovascular disease. SIRI, systemic inflammation response index. SII, Systemic immune-inflammation index.

| **Table S3. Survey-weighted cox proportional hazard results examining the association of SII with all-cause and cardiovascular disease mortality in the obese population. (SII was divided into quartiles, with the lowest group as the reference group)** | | | | | | |
| --- | --- | --- | --- | --- | --- | --- |
|  | Death | Crude  Model | Adjusted  Model 1 | Adjusted  Model 2 | Adjusted  Model 3 | Adjusted  Model 4 |
|  |  | HR (95%CI) | HR (95%CI) | HR (95%CI) | HR (95%CI) | HR (95%CI) |
|  |  | **All-cause mortality** | | | | |
| Q1 | 341 | Reference |  |  |  |  |
| Q2 | 403 | 0.87 (0.74-1.03) ^c^ | - | - | - | - |
| Q3 | 489 | 0.83 (0.71-0.98) ^c^ | - | - | - | - |
| Q4 | 726 | 1.08 (0.92-1.28) ^c^ | - | - | - | - |
| P for trend |  | 0.256 | - | - | - | - |
| Per SD increase |  | 1.12 (1.07-1.17) ^b^ | 1.10 (1.03-1.16) ^a^ | 1.09 (1.02-1.16) ^a^ | 1.09 (1.02-1.17) ^a^ | 1.09 (1.02-1.16) ^a^ |
|  |  | **Cardiovascular disease mortality** | | | | |
| Q1 | 121 | Reference |  |  |  |  |
| Q2 | 131 | 0.96 (0.69-1.32) ^c^ | - | - | - | - |
| Q3 | 130 | 0.83 (0.56-1.23) ^c^ | - | - | - | - |
| Q4 | 171 | 1.32 (0.97-1.81) ^c^ | - | - | - | - |
| P for trend |  | 0.104 | - | - | - | - |
| Per SD increase |  | 1.15 (1.06-1.24) ^b^ | 1.11 (1.10-1.12) ^a^ | 1.11 (1.03-1.20) ^a^ | 1.14 (1.04-1.26) ^a^ | 1.14 (1.04-1.26) ^a^ |

**If no significant association was observed, which indicated no independent association of SII with outcome. Subsequent outcomes were then not performed.**

**^a^ indicates p-value**<**0.05; ^b^ indicates p-value**<**0.001; ^c^ indicates p-value**≥**0.05.**

**Model 1** adjust age.

**Model 2** adjust **Model 1** plus other demographic variables including sex, race, education levels and poverty income ratio.

**Model 3** adjusted **Model 2** plus other parameters including body mass index, alanine aminotransferase, aspartate aminotransferase, and high-density lipoprotein cholesterol.

**Model 4** adjusted **Model 3** plus history of diseases including diabetes mellitus and hyperlipidemia.

| **Table S4.**  **Threshold-effect analysis on SIRI, SII, and all-cause and CVD mortality.** | | | | | |
| --- | --- | --- | --- | --- | --- |
|  | **Inflection-point (10^3^ cells/ml)** | **HR** | **95% CI** | **p-value** | **p for nonlinear** |
| **SIRI (1 unit increase)** | **All-cause Mortality** | 1.20^a^ | 1.13-1.28 | ＜0.001 | 0.492 |
|  | **CVD mortality** | 1.26^a^ | 1.14-1.40 | ＜0.001 | 0.039 |
|  | **＜2.27** | 1.50^a^ | 1.23-1.82 | ＜0.001 |  |
|  | **≥2.27** | 1.05^a^ | 0.85-1.31 | 0.642 |  |
| **SII (100 units increase)** | **All-cause Mortality** | 1.03^b^ | 1.02-1.05 | ＜0.001 | 0.152 |
|  | **CVD mortality** | 1.05^b^ | 1.02-1.08 | ＜0.001 | 0.027 |
|  | **＜246** | 2.26^b^ | 1.04-4.88 | 0.04 |  |
|  | **≥246** | 1.04^b^ | 1.02-1.07 | 0.002 |  |

^a^ indicated HR has been fully adjusted for the following variables: age, race, sex, poverty income ratio, body mass index, estimated glomerular filtration rate, alanine aminotransferase, total cholesterol, high-density lipoprotein cholesterol, cardiovascular diseases, diabetes mellitus, hypertension, hyperlipidemia, antihypertensives, glucose-lowering drugs, lipid-lowering drugs, smoking, and drinking.

^b^ indicated HR has been fully adjusted for the following variables: age, race, sex, poverty income ratio, education levels, body mass index, alanine aminotransferase, aspartate aminotransferase, high-density lipoprotein cholesterol, diabetes mellitus, and hyperlipidemia.

| **Table S5. Differences in the baseline characteristics of survivors and the death.** | | | |
| --- | --- | --- | --- |
|  | **Survivors** | **Death** | **P-value** |
| **Number** | 11067 | 1959 |  |
| **Age** | 44.6 ± 15.9 | 66.0 ± 13.6 | <0.001 |
| **PIR** | 2.4 ± 1.6 | 2.1 ± 1.4 | <0.001 |
| **BMI** | 35.8 ± 5.8 | 35.6 ± 5.5 | 0.163 |
| **HDL-C** | 1.2 ± 0.3 | 1.3 ± 0.4 | 0.004 |
| **TC** | 5.1 ± 1.1 | 5.1 ± 1.2 | 0.686 |
| **CKDEPISCR** | 99.2 ± 22.5 | 74.2 ± 24.5 | <0.001 |
| **ALT** | 29.0 ± 21.8 | 24.3 ± 16.2 | <0.001 |
| **AST** | 26.1 ± 16.4 | 25.6 ± 14.4 | 0.276 |
| **SIRI** | 1.2 ± 0.7 | 1.5 ± 1.0 | <0.001 |
| **Sex** |  |  | <0.001 |
| Female | 6235 (56.3%) | 962 (49.1%) |  |
| Male | 4832 (43.7%) | 997 (50.9%) |  |
| Race |  |  | <0.001 |
| Non-Hispanic white people | 4265 (38.5%) | 1037 (52.9%) |  |
| Non-Hispanic black people | 3011 (27.2%) | 466 (23.8%) |  |
| Mexican American | 2402 (21.7%) | 311 (15.9%) |  |
| Other races | 1389 (12.6%) | 145 (7.4%) |  |
| **Educational levels** |  |  | <0.001 |
| Less than 9th grade | 1149 (11.0%) | 436 (22.4%) |  |
| 9-11th grade/high school grade or equivalent | 4295 (41.2%) | 885 (45.5%) |  |
| College graduate or above | 4975 (47.7%) | 626 (32.2%) |  |
| **CVD** |  |  | <0.001 |
| No | 9585 (91.9%) | 1299 (66.5%) |  |
| Yes | 841 (8.1%) | 653 (33.5%) |  |
| **DM** |  |  | <0.001 |
| No | 8867 (80.1%) | 1112 (56.8%) |  |
| Yes | 2200 (19.9%) | 847 (43.2%) |  |
| **Hyperlipidemia** |  |  | <0.001 |
| No | 2315 (20.9%) | 315 (16.1%) |  |
| Yes | 8752 (79.1%) | 1644 (83.9%) |  |
| **Hypertension** |  |  | <0.001 |
| No | 5977 (54.0%) | 409 (20.9%) |  |
| Yes | 5090 (46.0%) | 1550 (79.1%) |  |
| **Smoke** |  |  | <0.001 |
| Never | 6065 (57.9%) | 842 (43.2%) |  |
| Former | 2370 (22.6%) | 745 (38.2%) |  |
| Current | 2048 (19.5%) | 361 (18.5%) |  |
| **Alcohol user** |  |  | <0.001 |
| Never | 1468 (15.2%) | 320 (17.8%) |  |
| Former | 1865 (19.3%) | 675 (37.6%) |  |
| Mild/Moderate | 2847 (29.4%) | 493 (27.5%) |  |
| Heavy | 3492 (36.1%) | 307 (17.1%) |  |
| **Antihypertensives** |  |  | <0.001 |
| No | 9885 (89.4%) | 1342 (68.6%) |  |
| Yes | 1177 (10.6%) | 613 (31.4%) |  |
| **Glucose-lowering drugs** |  |  | <0.001 |
| No | 9768 (88.3%) | 1379 (70.5%) |  |
| Yes | 1294 (11.7%) | 576 (29.5%) |  |
| **Lipid-lowering drugs** |  |  | <0.001 |
| No | 9293 (84%) | 1271 (65%) |  |
| Yes | 1769 (16%) | 684 (35%) |  |

**Categorical variables were expressed as count (percentage %).**

**Continuous variables were expressed as mean ± standard deviation.**

PIR, Poverty income ratio. ALT, Alanine aminotransferase. AST, Aspartate aminotransferase.TC, Total cholesterol. HDL, High-density lipoprotein cholesterol. CVD, cardiovascular diseases. eGFR, estimated glomerular filtration rate.

| **Table S6. Multivariate cox regression to assess the relationship between baseline variables and all-cause mortality.** | | | | |
| --- | --- | --- | --- | --- |
|  | **HR** | **95%CI low** | **95% high** | **P-value** |
| **SIRI** | 1.1845 | 1.1259 | 1.2462 | <0.0001 |
| **Age** | 1.0731 | 1.0671 | 1.0791 | <0.0001 |
| **Sex** | 1.4933 | 1.3252 | 1.6828 | <0.0001 |
| **Race** |  |  |  |  |
| Non-Hispanic black people | 0.9514 | 0.8323 | 1.0875 | 0.4650 |
| Mexican American | 0.7039 | 0.5995 | 0.8265 | <0.0001 |
| Other races | 0.7642 | 0.6219 | 0.9391 | 0.0105 |
| **Education levels** |  |  |  |  |
| 9-11th grade/high school grade or equivalent | 0.8992 | 0.7788 | 1.0382 | 0.1473 |
| College graduate or above | 0.8744 | 0.7445 | 1.0269 | 0.1017 |
| **PIR** | 0.8735 | 0.8393 | 0.9092 | <0.0001 |
| HDL | 1.0351 | 0.8822 | 1.2145 | 0.6722 |
| eGFR | 0.9898 | 0.9870 | 0.9927 | <0.0001 |
| ALT | 0.9976 | 0.9942 | 1.0011 | 0.1803 |
| **CVD** | 1.5736 | 1.4024 | 1.7658 | <0.0001 |
| **Diabetes mellitus** | 1.4165 | 1.2181 | 1.6471 | <0.0001 |
| **Hyperlipidemia** | 0.8532 | 0.7290 | 0.9869 | 0.0376 |
| **Hypertension** | 1.2009 | 1.0449 | 1.3802 | 0.0099 |
| **Smoke** |  |  |  |  |
| Former | 1.1684 | 1.0371 | 1.3164 | 0.0105 |
| Current | 1.9959 | 1.7191 | 2.3172 | <0.0001 |
| **Drinking** |  |  |  |  |
| Former | 1.0639 | 0.9116 | 1.2417 | 0.4320 |
| Mild/Moderate | 0.8503 | 0.7210 | 1.0028 | 0.0541 |
| Heavy | 0.8676 | 0.7182 | 1.0480 | 0.1406 |
| **Antihypertensives** | 1.0530 | 0.9388 | 1.1810 | 0.3778 |
| **Glucose-lowering drugs** | 1.1425 | 0.9698 | 1.3461 | 0.1112 |
| **Lipid-lowering drugs** | 0.9005 | 0.7982 | 1.0159 | 0.0885 |

**Variables marked in red indicate that they were selected for Nomogram plotting.**

PIR, Poverty income ratio. ALT, alanine aminotransferase. HDL, High-density lipoprotein cholesterol. CVD, cardiovascular diseases. SIRI, System Inflammation Response Index.

**Figure S1. The flow of the study chart.**


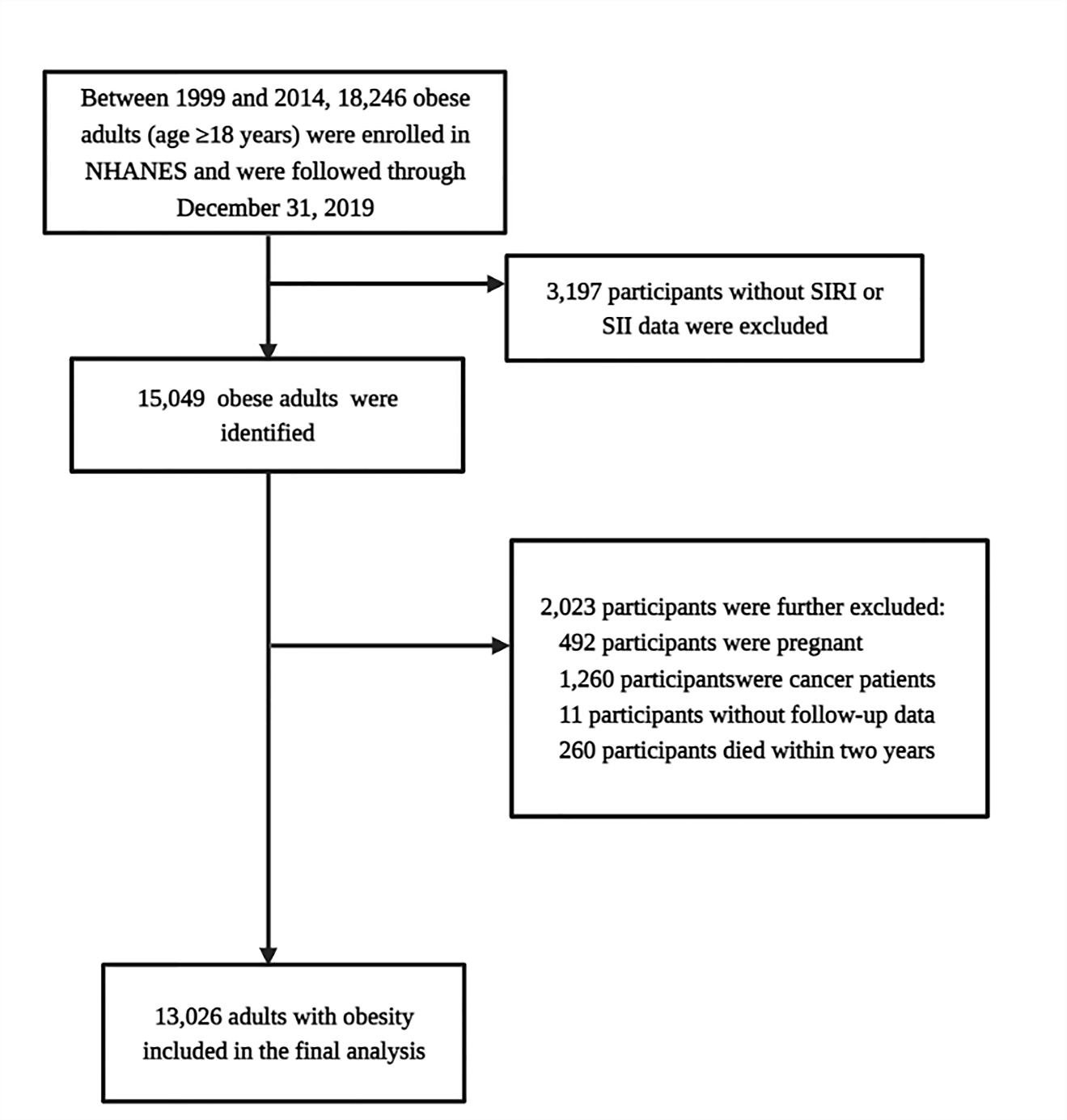


**Figure S2.**

1. Subgroup analyses of the associations (hazard ratios, 95% CIs) between SII increase and the risk of all-cause mortality. HRs indicate the increased risk of all-cause mortality for each standard deviation increase in SII.
2. Subgroup analyses of the associations (hazard ratios, 95% CIs) between SII increase and the risk of CVD mortality. HRs indicate the increased risk of CVD mortality for each standard deviation increase in SII.

HR has been fully adjusted by race, sex, poverty income ratio, education levels, body mass index, alanine aminotransferase, aspartate aminotransferase, high-density lipoprotein cholesterol, diabetes mellitus, and hyperlipidemia.


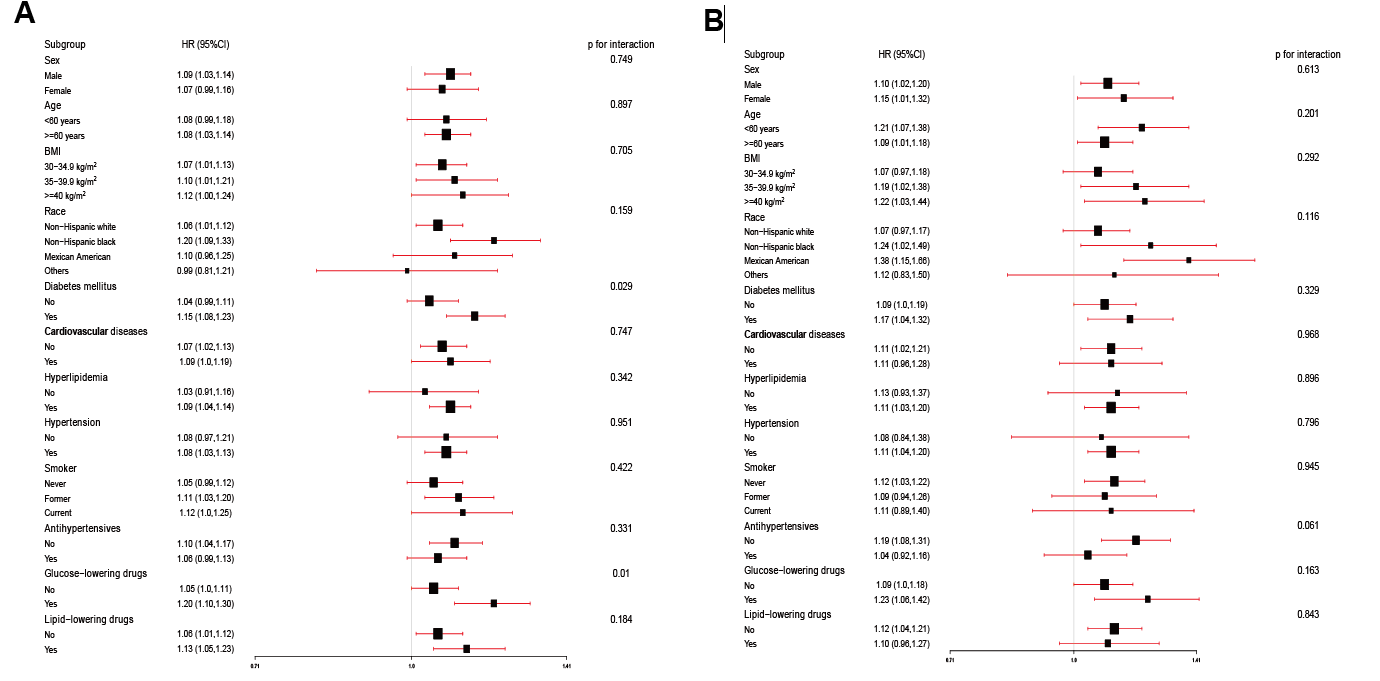


**Figure S3.**

1. **Smooth curve of the relationship between SII levels and the risk of all-cause mortality.**
2. **Smooth curve of the relationship between SII levels and the risk of CVD mortality.**


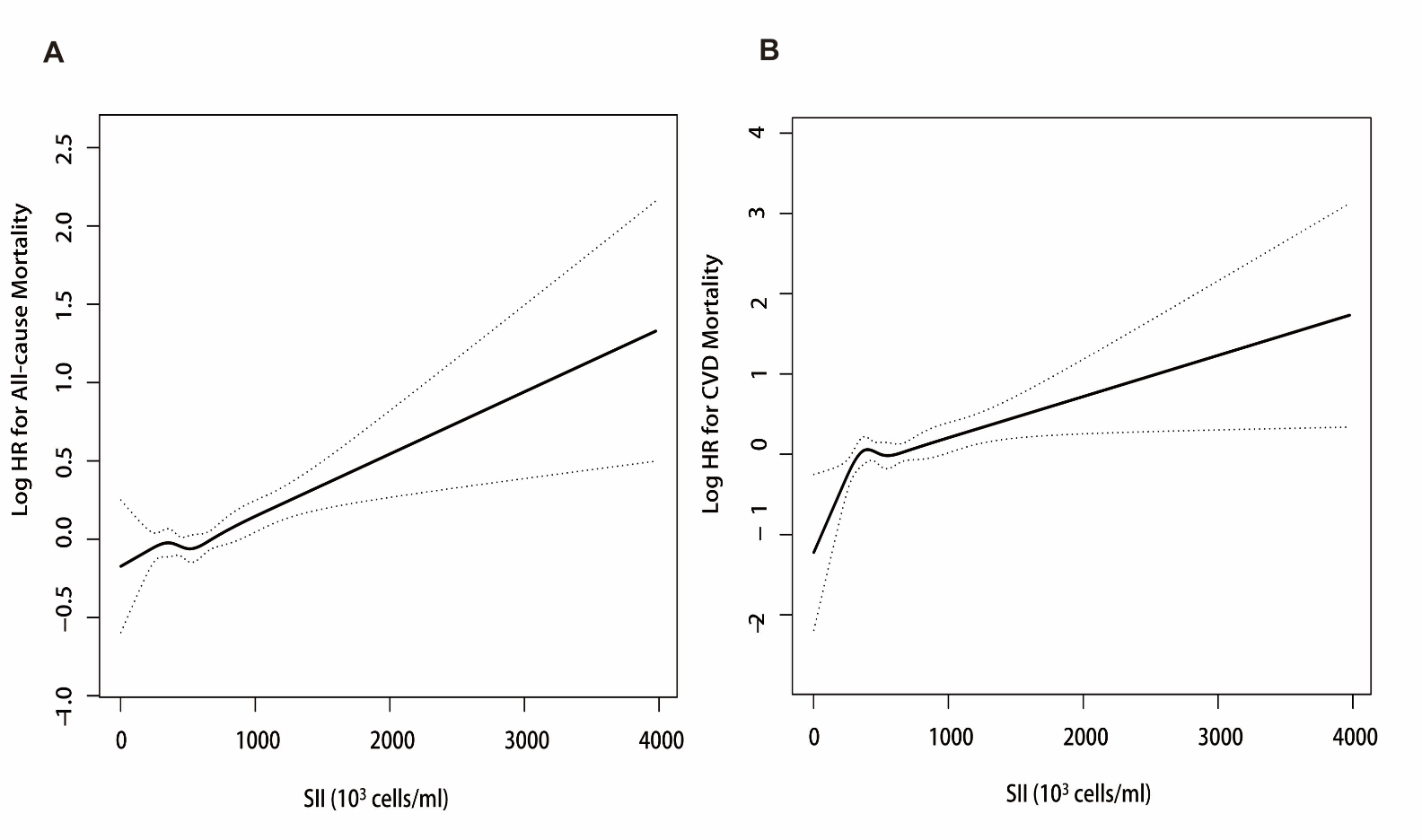
HR has been fully adjusted by race, sex, poverty income ratio, education levels, body mass index, alanine aminotransferase, aspartate aminotransferase, high-density lipoprotein cholesterol, diabetes mellitus, and hyperlipidemia.

**Figure S4.**

1. **Comparison of SIRI and SII predictions of all-cause mortality.**
2.
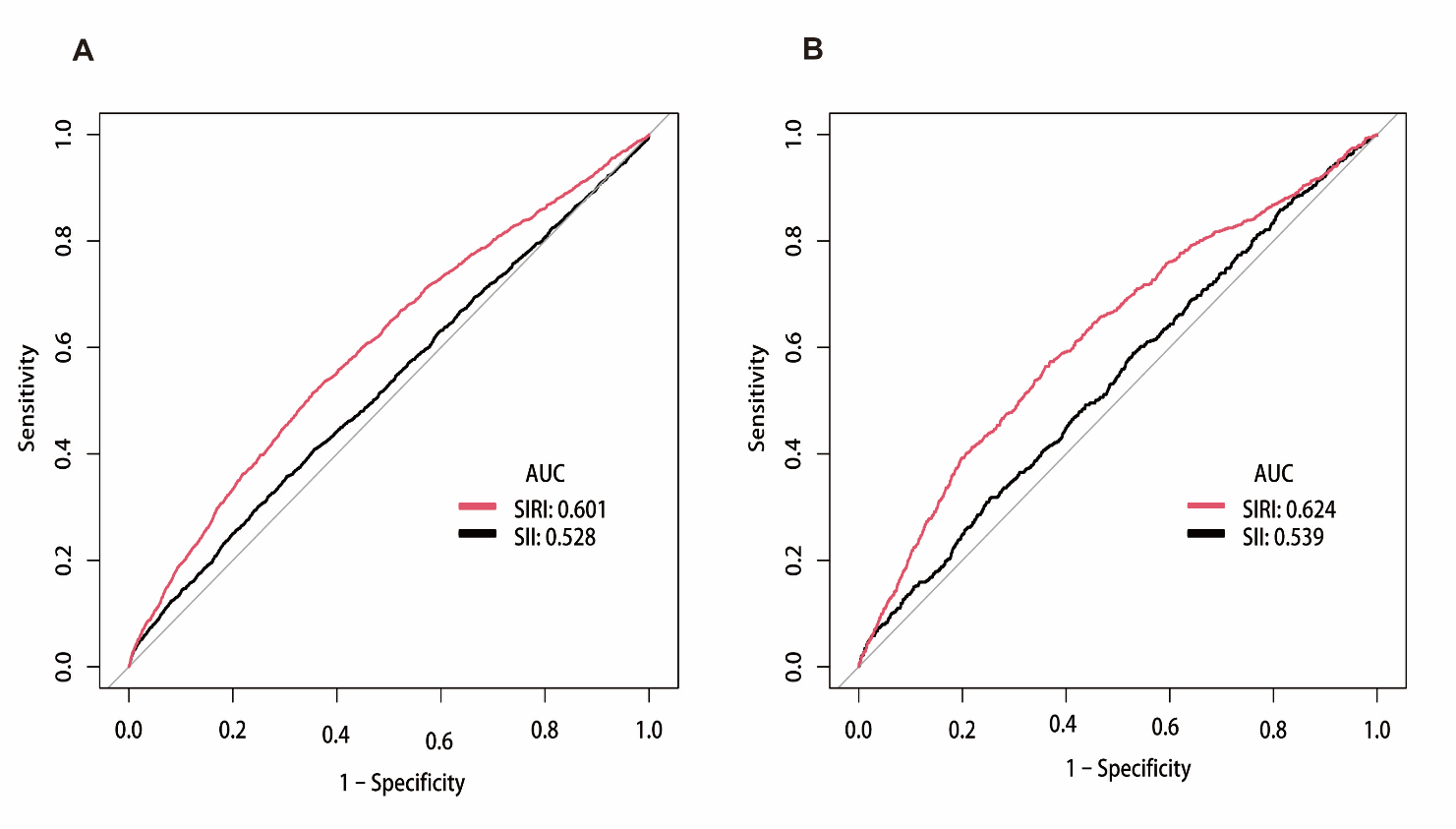
**Comparison of SIRI and SII predictions of CVD mortality.**

**Figure S5.** **Comparison of BMI levels between the major races and other multiracial groups.**

The bar graph is represented by the median (interquartile intervals). Differences between them were performed by the Mann-Whitney test (P-value<0.001). The main races include non-Hispanic white people, non-Hispanic black people, and Mexican Americans. The major race group BMI levels were: 34.1 (31.8-38.2) kg/m^2^. Multiple other races BMI levels were: 33.5 (31.5-36.9) kg/m^2^.


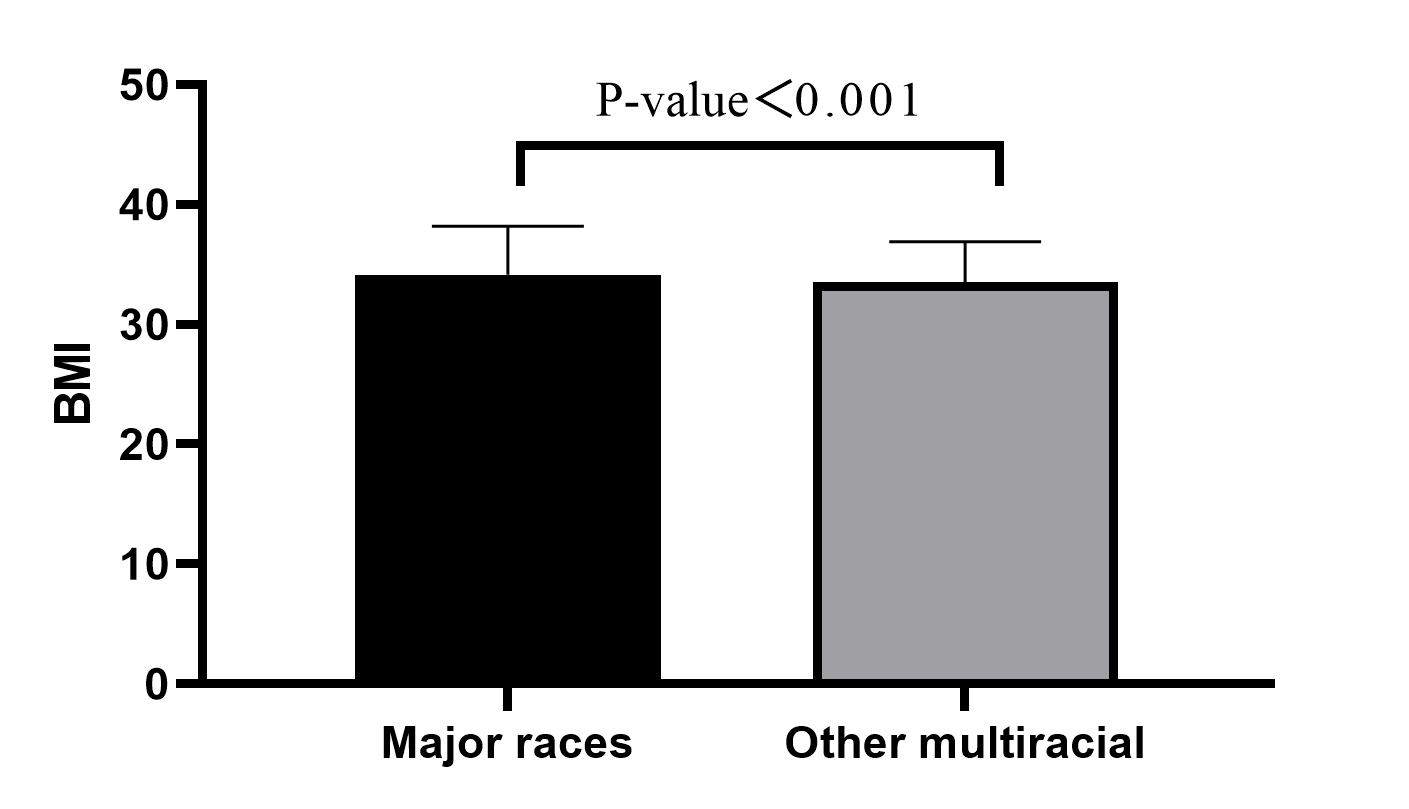


**Figure S6.** **Comparison of proportions of major races and other multiracial groups under different BMI categories.**

From left to right, in the three bar graphs, the percentage of other multiracial in the different BMI categories is 10.3%, 8.2% and 8.6%, respectively.

Differences in the proportion of races accounted for between the three BMI categories were tested using the survey-weighted Chi-square test (P-value=0.013).

**
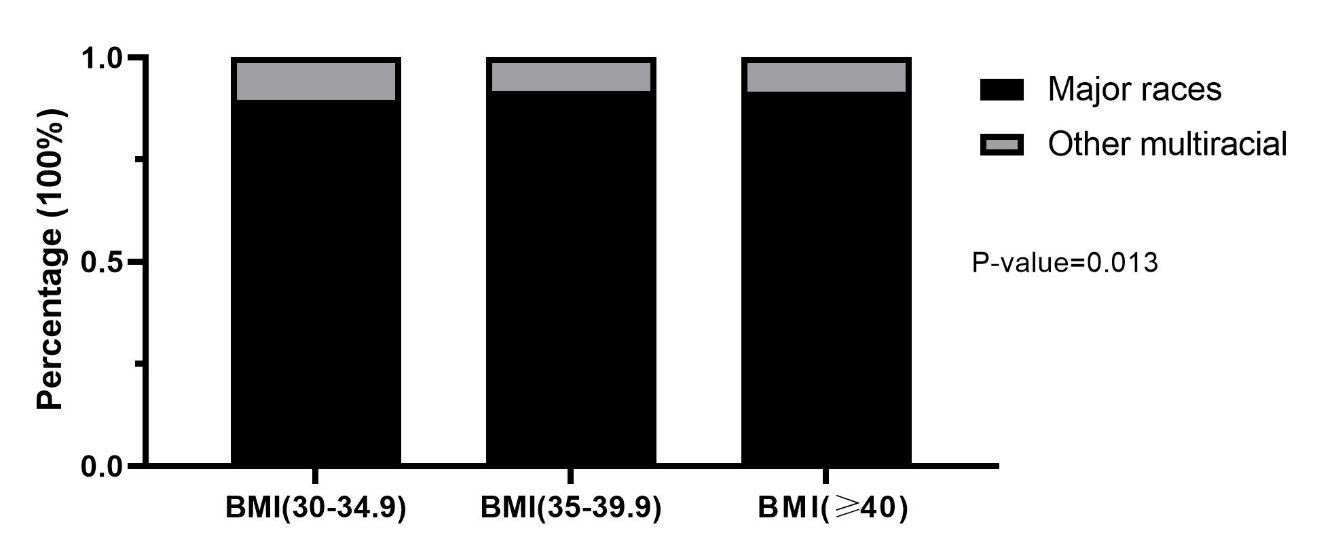
**

**Figure 7. Comparisons of SIRI and SII versus BMI for prediction of all-cause and CVD mortality.**


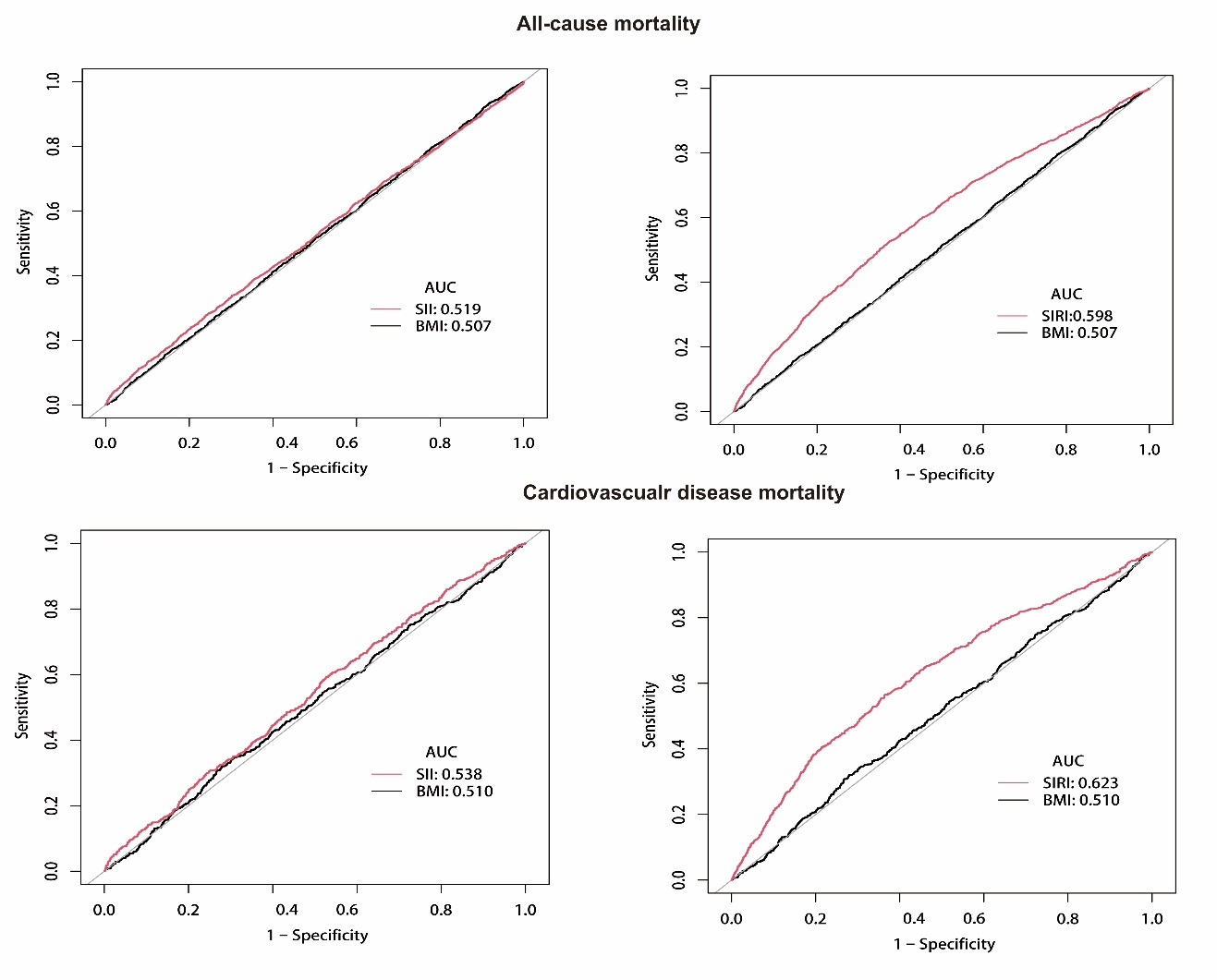

Supplement: Supplementary file 1 — Additional file 1: Figure S1. The flow of the study chart. Figure S2. A Subgroup analyses of the associations (hazard ratios, 95% CIs) between SII increase and the risk of all-cause mortality. HRs indicate the increased risk of all-cause mortality for each standard deviation increase in SII. B Subgroup analyses of the associations (hazard ratios, 95% CIs) between SII increase and the risk of CVD mortality. Figure S3. A Smooth curve of the relationship between SII levels and the risk of all-cause mortality. B Smooth curve of the relationship between SII levels and the risk of CVD mortality. Figure S4. A Comparison of SIRI and SII predictions of all-cause mortality. B Comparison of SIRI and SII predictions of CVD mortality. Figure S5. Comparison of BMI levels between the major races and other multiracial groups. Figure S6. Comparison of proportions of major races and other multiracial groups under different BMI categories. Figure S7. Comparisons of SIRI and SII versus BMI for prediction of all-cause and CVD mortality. Table S1. Survey-weighted baseline characteristics of the obese population in NHANES from 1999 to 2014 according to SII quartiles (N=13,026, representing 63,479,085 individuals with obesity). Table S2. Spearman correlation analysis of SIRI, SII, and baseline characteristics. Table S3. Survey-weighted cox proportional hazard results examining the association of SII with all-cause and cardiovascular disease mortality in the obese population. (SII was divided into quartiles, with the lowest group as the reference group). Table S4. Threshold-effect analysis on SIRI, SII, and all-cause and CVD mortality. Table S5. Differences in the baseline characteristics of survivors and the death. Table S6. Multivariate cox regression to assess the relationship between baseline variables and all-cause mortality. [file 13098_2023_1178_MOESM1_ESM.docx]
